# A comparative analysis of non-invasive respiratory support modalities in the treatment of acute hypercapnic respiratory failure: a network meta-analysis

**DOI:** 10.3389/fmed.2025.1594128

**Published:** 2025-07-08

**Authors:** Liyu Yan, Guishen Wu

**Affiliations:** Department of Critical Care Medicine, Zhongshan People’s Hospital, Zhongshan, China

**Keywords:** acute hypercapnic respiratory failure, non-invasive respiratory support, high-flow nasal cannula, non-invasive ventilation, conventional oxygen treatment

## Abstract

**Aim:**

The purpose of this study is to compare different non-invasive respiratory support methods for the treatment of acute hypercapnic respiratory failure (AHRF).

**Methods:**

The network meta-analysis was conducted based on studies from PubMed, Embase, the Cochrane Library, and Web of Science, from their inception to September 10, 2024. The outcomes was treatment failure, all-cause mortality, intubation, dyspnea score, length of stay in hospital, respiratory rate, arterial carbon dioxide partial pressure (PaCO_2_), and complications. The results of both direct and indirect comparisons were quantitatively assessed using weighted mean differences or relative risks with their respective 95% confidence intervals, and graphically depicted in forest plots. Additionally, the rank probabilities were presented, demonstrating the likelihood of each non-invasive respiratory support method being the most effective across various measured outcomes.

**Results:**

Nineteen studies (2,022 participants) were included. The results indicated that the probability of treatment failure with face mask non-invasive ventilation (NIV) was lower than that of high-flow nasal cannula (HFNC) (RR: 1.42, 95% CI: 1.06, 1.93) and conventional oxygen treatment (COT) (RR: 1.87, 95% CI: 1.16, 3.03). Face mask NIV demonstrated superior performance in dyspnea score and PaCO_2_ compared to HFNC, helmet NIV, and COT. The length of stay in the hospital for face mask NIV was relatively longer compared to HFNC (WMD: −0.73, 95% CI: −1.35, −0.10) and COT (WMD: −1.09, 95% CI: −2.00, −0.18), and the probability of complications was higher than with HFNC. The rank probability suggested that COT had the lowest likelihood of intubation and all-cause mortality, while helmet NIV may have the best effect on improving respiratory rate.

**Conclusion:**

Concerning treatment failure, dyspnea score, and PaCO_2_ improvement in patients with AHRF, face mask NIV may outperform other methods. For selected patients with AHRF, face mask NIV might be considered for potential first-line method. This study provides a certain level of evidence-based support for the management and treatment of AHRF, but more research is still needed in the future to determine the optimal non-invasive respiratory support method for treating patients with AHRF. In clinic, the efficacy of face mask NIV for better outcomes in patients with AHRH still requires validation.

## Introduction

Acute hypercapnic respiratory failure (AHRF) is a life-threatening clinical pulmonary condition characterized primarily by alveolar hypoventilation resulting in an arterial carbon dioxide partial pressure (PaCO_2_) of ≥45 mmHg and accompanied by acidosis with a pH less than 7.35 (1, 2). In some cases, AHRF is accompanied by hypoxemia, defined as an arterial oxygen partial pressure (PaO_2_) less than 60 mmHg (3). AHRF imposes a significant physiological health burden on patients. For instance, hypercapnic acidosis can diminish the migration of neutrophils to the site of inflammation and inhibit phagocytic activity, thereby disrupting immune mechanisms (4). Hypercapnic acidosis may also inhibit cardiac contractility and decrease systemic vascular resistance, influencing the normal functioning of the heart (4, 5). Moreover, AHRF is associated with high morbidity and mortality (6). Studies have reported that up to 19% of AHRF patients die after receiving treatment (7).

Currently, non-invasive respiratory support is the most usual method for treating AHRF. The central principle involves providing respiratory assistance to patients without the need for endotracheal intubation or tracheostomy (8). Common approaches include conventional oxygen treatment (COT), non-invasive ventilation (NIV), and high-flow nasal cannula oxygen therapy (HFNC) (9). NIV is primarily achieved by utilizing a ventilator to provide positive-pressure ventilation (10). In the European Respiratory Society/American Thoracic Society guidelines, NIV is strongly recommended for acidosis patients with a pH range of 7.25–7.35 (11). NIV has historically been the first-line treatment for patients with hypercapnia (11, 12). HFNC, an oxygen delivery system, a novel approach introduced in the last decade, may work by reducing anatomical dead space and improving mucociliary clearance (13, 14). HFNC is frequently utilized in the treatment of acute hypoxemic respiratory failure, and an increasing number of studies suggest that it may serve as an effective therapy for AHRF (14, 15). Existing meta-analyses have compared the therapeutic efficacy of the aforementioned methods in treating AHRF. For instance, a previous meta-analysis demonstrated that HFNC was effective and safe for the treatment of AHRF, and in patients with a pH less than 7.30, NIV was associated with a lower risk of treatment crossover compared to HFNC (3). However, Ovtcharenko’s et al. (16) survey conducted among patients with AHRF did not determine which was more effective between HFNC and NIV. Yet, given the conflicting findings from these studies and the fact that the majority of meta-analyses conducted have been conventional direct comparisons without considering the potential for indirect comparisons among various approaches, it is still uncertain which treatment method is the most beneficial for patients suffering from AHRF (2, 3, 16). Network meta-analysis (NMA) facilitates a more comprehensive estimation of three or more intervention efficacy by integrating both direct and indirect evidence, thereby enabling the ranking of various interventions, including those without prior direct comparisons, to identify the most effective treatment options (17).

Herein, in order to further investigate the effects of various non-invasive respiratory support methods on AHRF and to provide additional clinical reference information, an NMA was conducted. This analysis compared the therapeutic efficacy of different non-invasive respiratory support methods in adult patients with AHRF.

## Material and method

This systematic review and meta-analysis were conducted in strict accordance with the preferred reporting items for systematic reviews and meta-analyses (PRISMA) statements (18).

### Retrieval strategy

**Figure 1:**
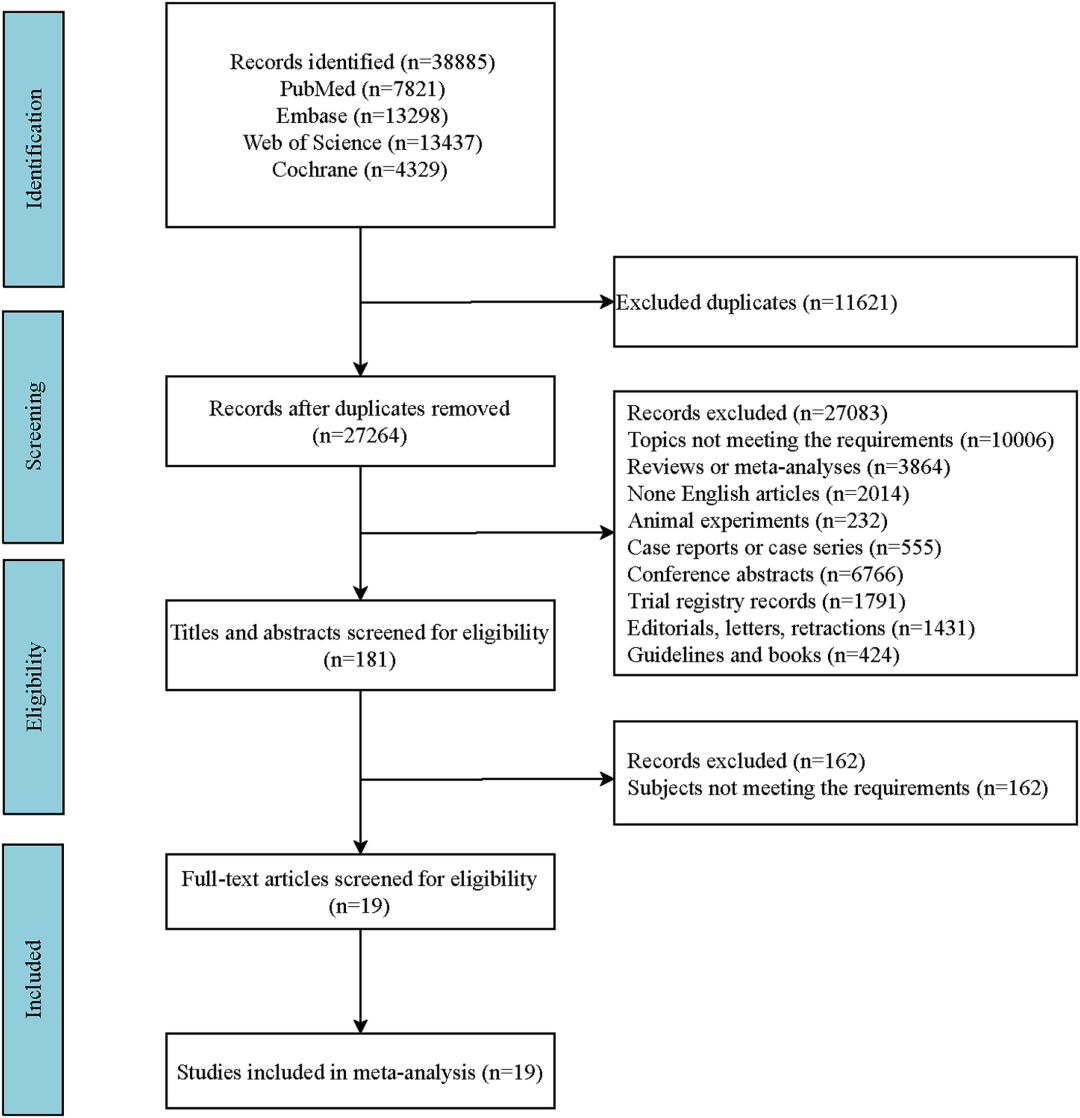
Flowchart of literature search selection.

A literature search was conducted across four English-language databases: PubMed, Embase, the Cochrane Library, and Web of Science. The specific search date was September 10, 2024. The Supplementary material include the detailed English search terms and the search formula employed in PubMed (Supplementary Table S1). A comprehensive outline of the search strategies is depicted in the flowchart (Figure 1). The retrieved literature records were imported into EndNote X20 software for management. After eliminating duplicate publications, the initial screening of the literature was conducted by reviewing titles and abstracts in accordance with the predefined inclusion and exclusion criteria. Subsequently, full-text articles were reviewed to exclude those that did not meet the requirements, and the remaining eligible articles were included in this study. Consequently, full-text articles of the included studies were reviewed to extract relevant data. The specific data items extracted included: author(s), publication year, country, study design, intervention(s), sample size, age, and relevant indicators of outcomes.

### Inclusion and exclusion criteria

Based on the patient intervention comparison outcomes (PICO) principles, the inclusion criteria for this systematic review were established as follows: (1) Study population: Adult patients (age ≥18 years) with AHRF, presenting with a pH <7.35 or PaCO_2_ >45 mmHg. (2) Interventions and comparisons: Interventions: HNFC, facemask NIV, helmet NIV, and COT; Comparisons: Direct or indirect comparisons between the aforementioned methods. (3) Outcomes: Treatment failure, all-cause mortality, intubation, dyspnea score, length of hospital stay (days), respiratory rate (breaths per minute), PaCO_2_ (mmHg), and complications. (4) Study types: Randomized controlled trials (RCTs) and cohort studies. Exclusion criteria: The articles were animal experimental studies, retracted publications, reviews, meta-analyses, case reports, case series, conference abstracts, editorial materials, letters, trial registry records, guidelines, books, non-English literature, and documents that did not pertain to the topic under investigation.

### Methods of literature quality evaluation

For RCTs, the Cochrane risk of bias assessment tool was employed (19). This tool evaluates the included studies based on seven criteria: “Random sequence generation,” “Allocation concealment,” “Blinding of participants and personnel,” “Blinding of outcome assessment,” “Incomplete outcome data,” “Selective reporting,” and “Other bias.” For cohort studies, a modified Newcastle–Ottawa Scale (NOS) was used, comprising three main items: “selection,” “comparability,” and “outcome.” Each study can receive up to one “star” for each item under “selection” and “outcome,” and up to two “stars” for the item under “comparability” (20). The total score ranges from 0 to 9, with 0–3 stars indicating low quality, 4–6 stars indicating moderate quality, and 7–9 stars indicating high quality.

Additionally, the grading of recommendations, assessment, development, and evaluation (GRADE) approach was utilized to grade the quality of evidence in the NMA (21). The evidence quality was assessed across five domains: study limitations, consistency of results, directness (generalizability), precision, and publication bias. The GRADE system classifies evidence into four levels: high, moderate, low, and very low. This study employed the GRADE pro GDT online tool to create the GRADE evidence profiles (22).

### Definition of outcomes

Treatment failure was defined as the need for invasive ventilation or a change in respiratory support modality, and the dyspnea score was assessed using the modified 0–10 Borg scale. Complications were defined as the occurrence of any conditions unrelated to the primary disease, such as skin erosion, rhinitis, conjunctivitis, gastric distension, pneumothorax, arrhythmia, and cardiac infarction. The measurement time points for outcomes were as follows: for PaCO_2_ and respiratory rate, the longest time point was before discharge. For the dyspnea score, the longest time point was 72 h. For all-cause mortality, the longest time point was 90 days. Due to limitations in the literature data, precise time points for tracheal intubation and treatment failure were not provided.

### Statistical analysis

NMA was conducted within a Bayesian framework using the Monte Carlo Markov Chain model, with a model chain length of 4, an initial iteration of 20,000, and subsequent updates of 50,000 iterations with a step size of 1. Heterogeneity was assessed using the *I*^2^ statistic, where *I*^2^ < 25, 25–50%, and >50% indicate low, moderate, and high heterogeneity, respectively. Consistency, a key assumption in NMA, was evaluated by comparing the deviance information criterion (DIC) between the consistency and inconsistency models; a smaller DIC difference indicates a better fit, with a difference of 5 or less suggesting that the data essentially meet the consistency assumption. Network evidence plots were constructed for each outcome measure. Weighted mean differences (WMDs) with 95% confidence intervals (CIs) were reported for dyspnea score, length of hospital stay, respiratory rate, and PaCO_2_; relative risk (RR) values with 95% CIs were reported for treatment failure, intubation, all-cause mortality, and complications. Forest plots presented both direct and indirect comparisons of WMDs or RRs with 95% credible intervals. Ranking probability plots were used to predict the relative merits of each non-invasive respiratory support method, with bar charts representing the probability of each intervention being ranked at the nth position, and the x-axis indicating the relevant rank. The quality of randomized controlled studies was assessed using RevMan 5.3, generating summary graphs of risk of bias. All analyses were performed using Stata 15.1 software and the Gemtc package (version 1.0.2) in R version 4.2.3.

## Results

### Literature identification and selection

Following the search strategy, a total of 38,885 records were identified. After removing 11,621 duplicates, 27,264 records remained. Further screening based on the predefined inclusion and exclusion criteria by reviewing titles and abstracts led to the exclusion of 27,083 records, leaving 181 articles. Upon full-text review, 161 articles that did not meet the requirements of study participants were excluded, resulting in the inclusion of 19 studies. A list of the literature for specific analysis is provided in the Supplementary material. The included studies consisted of four cohort studies and 15 RCTs. A more detailed screening process of the literature is shown in Figure 1.

### Baseline characters of included literature

Table 1 presents the descriptive features of the studies that were incorporated into the NMA. A total of 2,022 patients were included in this study. Among them, 699 patients received NIV via face mask, 890 patients were treated with HFNC, 101 patients used helmet NIV, and 332 patients received COT. The studies included in this research spanned a publication period of 15 years (2009–2024), with the majority conducted in Asia.

**Table 1:** Characteristics of the studies included in this network meta-analysis.

Author	Year	Country	Study design	Setting	Group	*N*	Male/Female	Age, years	Respiratory frequency, min	pH	PaCO_2_, mmhg	PaO_2_/FiO_2_, mmhg	APACHE II score	SAPS II score	Outcome
Tan	2024	China	RCT	ICU	HFNC	113	71/42	73 (65–78)^b^	28 (25–30)^b^	7.31 (7.29–7.33)^b^	63 (59–68)^b^	175 (167–199)^b^	14 (11–17)^b^	32 (26–37)^b^	Length of stay in hospital, respiratory rate, PaCO_2_, dyspnea score, treatment failure, intubation, all-cause mortality
					Face mask noninvasive ventilation	112	62/50	69 (63–76)^b^	29 (26–32)^b^	7.30 (7.28–7.32)^b^	61 (58–65)^b^	184 (167–202)^b^	12 (10–16)^b^	29 (26–34)^b^	
Pisani	2015	Italy	RCT	ICU	Helmet noninvasive ventilation	39	NR	78.36 ± 10.58^a^	30.82 ± 8.10^a^	7.27 ± 0.05^a^	72.58 ± 14.87^a^	193.33 ± 50.74^a^	NR	35.41 ± 10.36^a^	Respiratory rate, dyspnea score
					Face mask noninvasive ventilation	41		78.48 ± 7.75^a^	32.88 ± 8.75^a^	7.26 ± 0.06^a^	74.45 ± 15.25^a^	194.05 ± 67.89^a^		35.68 ± 10.51^a^	
Golmohamad	2022	Australia	Cohort study	Thoracic medicine unit	HFNC	22	10/12	72 ± 14^a^	NR	7.33 ± 0.05^a^	60 ± 11^a^	NR	NR	NR	All-cause mortality
					Face mask noninvasive ventilation	42	24/18	67 ± 14^a^	NR	7.27 ± 0.09^a^	74 ± 16^a^				
Papachatzakis	2020	Greece	RCT	ICU	HFNC	20	10/10	76.0 ± 13.4^a^	21.3 ± 8.7^a^	7.4 ± 0.1^a^	60.4 ± 9.9^a^	NR	21.6 ± 8.9^a^	NR	Length of stay in hospital, respiratory rate, PaCO_2_, all-cause mortality
					Face mask noninvasive ventilation	20	9/11	78.1 ± 8.1^a^	26.6 ± 5.3^a^	7.4 ± 0.1^a^	62.1 ± 10.3^a^		19.3 ± 6.1^a^		
Cuvelier	2009	France	RCT	ICU	Helmet noninvasive ventilation	17	12/5	77.8 ± 8.9^a^	24.3 ± 4.2^a^	7.28 ± 0.08^a^	10.6 ± 2.5^a^	209.9 ± 72.0^a^	NR	30.0 ± 8.8^a^	Length of stay in hospital, intubation, all-cause mortality, complications
					Face mask noninvasive ventilation	17	13/4	70.0 ± 12.0^a^	26.3 ± 4.7^a^	7.31 ± 0.04^a^	9.2 ± 2.1^a^	231.7 ± 69.0^a^		27.8 ± 7.4^a^	
Lee	2018	Korea	Cohort study	Respiratory center	HFNC	44	28/16	73 (68–79)^b^	24 (20–28)^b^	7.32 ± 0.28^a^	56.4 ± 10.1^a^	134.8 ± 7.3^a^	NR	NR	Intubation, all-cause mortality
					Face mask noninvasive ventilation	44	29/15	77 (71–80)^b^	24 (22–29)^b^	7.31 ± 0.29^a^	52.6 ± 8.8^a^	134.5 ± 7.5^a^			
Li	2020	China	RCT	General wards of respiratory departments	HFNC	160	101/59	68.4 ± 7.7^a^	21.0 ± 1.7^a^	7.38 ± 0.03^a^	54.9 ± 7.1^a^	NR	15.8 ± 6.5^a^	NR	Length of stay in hospital, respiratory rate, PaCO_2_, treatment failure, intubation, all-cause mortality
					COT	160	106/54	68.3 ± 6.9^a^	21.1 ± 1.9^a^	7.39 ± 0.04^a^	54.2 ± 6.0^a^		14.7 ± 6.0^a^		
Marjanovic	2020	France	RCT	Emergency department	HFNC	12	7/5	91 (77–94)^b^	34 (27–41)^b^	7.30 (7.20–7.36)^b^	50 (49–61)^b^	NR	NR	NR	Respiratory rate, PaCO_2_, intubation
					Face mask noninvasive ventilation	15	3/12	82 (79–92)^b^	29 (26–36)^b^	7.29 (7.21–7.35)^b^	60 (48–71)^b^				
Wang	2023	China	Cohort study	ICU	HFNC	44	33/11	78.64 ± 8.7^a^	23 ± 4^a^	7.33 ± 0.06^a^	70.2 ± 19.7^a^	197.3 ± 58.7^a^	16 ± 4^a^	32 ± 5^a^	Treatment failure, intubation, all-cause mortality
					Face mask noninvasive ventilation	44	32//12	80.2 ± 8.3^a^	22 ± 5^a^	7.33 ± 0.10^a^	70.0 ± 19.4^a^	202.6 ± 48.2^a^	17 ± 4^a^	34 ± 7^a^	
Doshi	2020	United States	RCT	Emergency department	HFNC	34	15/19	65.0 (56–73)^b^	32 (28–36)^b^	7.33 (7.24–7.40)^b^	56 (48–72)^b^	NR	31.0 (28–34)^b^	NR	Respiratory rate, PaCO_2_, dyspnea score, treatment failure, intubation
					Face mask noninvasive ventilation	31	15/16	59 (59–70)^b^	28 (24–32)^b^	7.32 (7.26–7.39)^b^	64.6 (48–91)^b^		29 (26–34)^b^		
Cortegiani	2020	Italy	RCT	Emergency department, ICU, or respiratory unit	HFNC	40	21/19	74 ± 13^a^	27 ± 7^a^	7.30 ± 0.03^a^	73.7 ± 12.8^a^	203.2 ± 45.5^a^	NR	30 ± 9^a^	Length of stay in hospital, respiratory rate, PaCO_2_, dyspnea score, all-cause mortality
					Face mask noninvasive ventilation	39	19/20	77 ± 12^a^	28 ± 7^a^	7.29 ± 0.03^a^	72.0 ± 13.0^a^	222.4 ± 71.0^a^		33 ± 10^a^	
Yoo	2016	Korea	Cohort study	ICU	HFNC	34	18/16	62.1 ± 16.8^a^	20.9 ± 6.0^a^	7.48 ± 0.07^a^	38.2 ± 6.6^a^	188.9 ± 73.8^a^	19.7 ± 4.1^a^	NR	Intubation, all-cause mortality
					Face mask noninvasive ventilation	39	25/14	62.9 ± 16.1^a^	22.8 ± 5.2^a^	7.40 ± 0.1^a^	48.2 ± 17.8^a^	190.6 ± 82.8^a^	19.2 ± 3.9^a^		
Ketan	2024	India	RCT	ICU	HFNC	30	20/10	65.3 ± 7.79 ^a^	16.27 ± 2.05^a^	7.42 ± 0.04^a^	48 ± 7.91^a^	244.67 ± 71.95^a^	NR	NR	Length of stay in hospital, treatment failure, intubation, all-cause mortality
					Face mask noninvasive ventilation	32	22/10	65.38 ± 9.76^a^	17.75 ± 2.23^a^	7.42 ± 0.03^a^	47 ± 4.81^a^	267.81 ± 47.98^a^			
Tan	2020	China	RCT	ICU	HFNC	44	27/17	68.4 ± 9.3^a^	18 (16–23)^b^	7.48 (7.42–7.51)^b^	50.5 (48–57.8)^b^	239.2 ± 47.0^a^	14 (11–18.8)^b^	27 (22–32.8)^b^	Length of stay in hospital, respiratory rate, PaCO_2_, dyspnea score, treatment failure, intubation, all-cause mortality
					Face mask noninvasive ventilation	42	23/19	71.4 ± 7.8^a^	21 (16–26)^b^	7.45 (7.40–7.49)^b^	53 (48.8–61.3)^b^	229.3 ± 42.0^a^	13 (10.8–16)^b^	30 (24–34.8)^b^	
Cong	2019	China	RCT	ICU	HFNC	84	48/36	66.91 ± 7.38^a^	NR	7.25 ± 0.08^a^	72.11 ± 16.31^a^	NR	NR	NR	Length of stay in hospital, PaCO_2_, complications
					Face mask noninvasive ventilation	84	50/34	67.88 ± 8.38^a^		7.27 ± 0.09^a^	72.91 ± 16.41^a^				
Çakir	2015	Turkey	RCT	ICU	Face mask noninvasive ventilation	23	15/8	64.3 ± 10.1^a^	27.1 ± 5.6^a^	7.28 ± 0.04^a^	69.7 ± 10.0^a^	NR	16.87 ± 4.78^a^	NR	Intubation, all-cause mortality, complications
					Helmet noninvasive ventilation	25	19/6	69.5 ± 7.41^a^	24.4 ± 6.3^a^	7.30 ± 0.03^a^	65.4 ± 12.0^a^		16.48 ± 3.89^a^		
Pantazopoulos	2024	Greece	RCT	Emergency and respiratory departments	HFNC	51	34/17	72.37 ± 9.18^a^	30 (6)^b^	7.30 (0.03)^b^	60.80 (10)^b^	237.78 (58.27)^b^	NR	NR	Treatment failure, intubation, all-cause mortality, complications
					Face mask noninvasive ventilation	54	41/13	73.26 ± 9.50^a^	32.50 (10)^b^	7.29 (0.05)^b^	65 (13)^b^	238.10 (43.19)^b^			
Xia	2022	China	RCT	General respiratory wards	HFNC	158	140/18	70.0 (65.0–75.0)^b^	21.0 (20.0–23.0)^b^	7.40 (7.37–7.42)^b^	50.4 (47.3–56.3)^b^	NR	10.0 (7.0–13.0)^b^	NR	Length of stay in hospital, respiratory rate, PaCO_2_, dyspnea score, treatment failure, intubation, all-cause mortality
					COT	172	137/35	69.0 (63.5–74.5)^b^	21.0 (20.0–23.0)^b^	7.40 (7.37–7.43)^b^	51.7 (47.6–58.0)^b^		10.0 (7.0–13.5)^b^		
Antonaglia	2011	Italy	RCT	ICU	Face mask noninvasive ventilation	20	NR	71 ± 7^a^	32 (29–34)^b^	7.20 ± 0.06^a^	77 ± 6.8^a^	169 ± 33.9^a^	23 (20–27)^b^	NR	Respiratory rate, PaCO_2_, intubation
					Helmet noninvasive ventilation	20		69 ± 8^a^	33 (30–35)^b^	7.21 ± 0.05^a^	79 ± 7.2^a^	175 ± 28^a^	22 (20–26)^b^		

HFNC, high flow nasal cannula; ICU, intensive care unit; APACHE II, Acute Physiology and Chronic Health Evaluation II; SAPS II, Simplified Acute Physiology Score II; PaCO_2_, arterial carbon dioxide partial pressure; FiO_2_, fraction of inspiration O_2_; NR, non-reported; RCT, randomized controlled trails; COT, conventional oxygen therapy.

a Mean ± SD.

b Median (IQR).

### Literature quality evaluation

The results of the four cohort studies evaluated using the modified NOS are presented in Supplementary Table S2, with one study rated as high quality and the remaining three as moderate quality. The summary of bias risk assessment for the 15 RCT articles is depicted in Supplementary Figure 1. Five studies had an unclear risk regarding the generation of random sequences and allocation concealment. All included studies were identified as having a high risk of bias due to a lack of blinding of participants and personnel. Based on incomplete outcome data and selective reporting, all trials were assessed to be at low risk. We summarized the GRADE deterministic results in Supplementary Table S3.

## Results of non-invasive respiratory support in the treatment of AHRF

### Treatment failure

**Figure 2:**
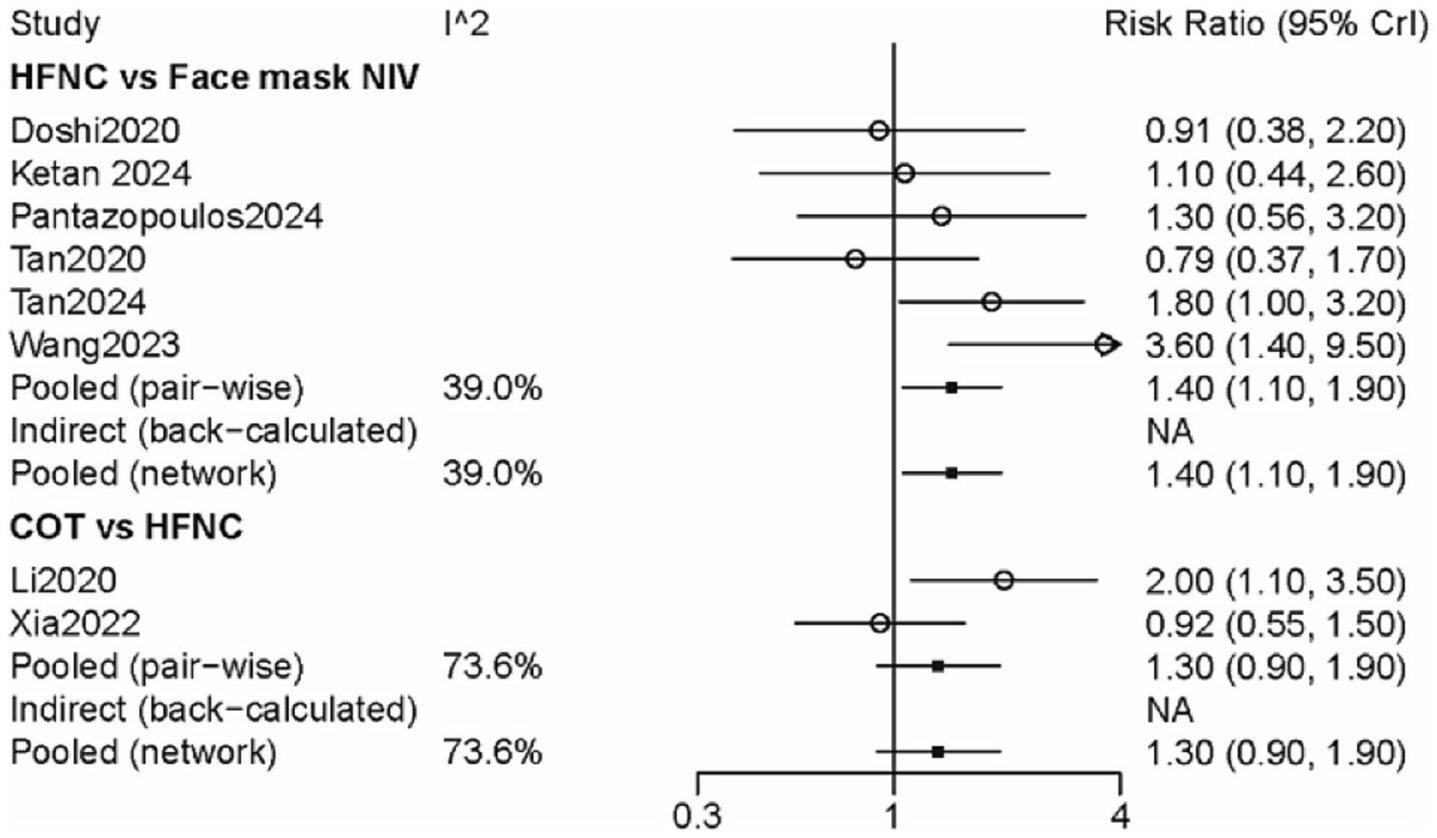
Forest plot on meta-analysis of treatment failure after treatment of AHRF with different noninvasive respiratory support methods.

Eight studies with 1,281 patients assessed treatment failure, and 3 interventions were compared (Supplementary Figure 2). The treatment failure rate in the HFNC group was higher than that in the face mask NIV group, with an RR of 1.40 (95% CI: 1.10, 1.90) (Figure 2). Table 2 demonstrated that the treatment failure rates for both the HFNC group (RR: 1.42, 95% CI: 1.06, 1.93) and the COT group (RR: 1.87, 95% CI: 1.16, 3.03) were higher than that of the face mask NIV group. The ranking probability plot indicated that the treatment failure rate in the face mask NIV group was the lowest among the three intervention methods (Table 3).

**Table 2:** League table of different non-invasive respiratory support methods for each outcome.

**Outcomes**	Rate	Rate	Rate	Rate
**Treatment failure**				
	Face mask NIV	HFNC	COT	
Face mask NIV	Face mask NIV	1.42 (1.06, 1.93)	1.87 (1.16, 3.03)	
HFNC	0.7 (0.52, 0.94)	HFNC	1.31 (0.91, 1.91)	
COT	0.54 (0.33, 0.86)	0.76 (0.52, 1.1)	COT	
**Intubation**				
	Face mask NIV	Helmet NIV	HFNC	COT
Face mask NIV	Face mask NIV	0.43 (0.14, 1.09)	0.97 (0.67, 1.4)	0.23 (0.01, 2.15)
Helmet NIV	2.32 (0.92, 7.01)	Helmet NIV	2.26 (0.83, 7.2)	0.53 (0.02, 6.45)
HFNC	1.03 (0.71, 1.5)	0.44 (0.14, 1.2)	HFNC	0.24 (0.01, 2.14)
COT	4.37 (0.47, 132.31)	1.88 (0.15, 63.15)	4.24 (0.47, 126.03)	COT
**All-cause mortality**				
	Face mask NIV	Helmet NIV	HFNC	COT
Face mask NIV	Face mask NIV	0.95 (0.11, 8.7)	0.99 (0.68, 1.44)	0.9 (0.23, 3.45)
Helmet NIV	1.05 (0.11, 9.41)	Helmet NIV	1.04 (0.11, 9.69)	0.95 (0.07, 12.15)
HFNC	1.01 (0.7, 1.48)	0.96 (0.1, 9.03)	HFNC	0.91 (0.25, 3.3)
COT	1.11 (0.29, 4.27)	1.05 (0.08, 14.1)	1.1 (0.3, 4.01)	COT
**Complications**				
	Face mask NIV	Helmet NIV	HFNC	
Face mask NIV	Face mask NIV	0.58 (0.3, 1.04)	0.6 (0.44, 0.8)	
Helmet NIV	1.71 (0.96, 3.35)	Helmet NIV	1.03 (0.53, 2.13)	
HFNC	1.66 (1.24, 2.29)	0.97 (0.47, 1.88)	HFNC	
**Dyspnea score**				
	Face mask NIV	Helmet NIV	HFNC	COT
Face mask NIV	Face mask NIV	0.98 (0.09, 1.87)	0.54 (0.26, 0.82)	0.71 (0.31, 1.11)
Helmet NIV	-0.98 (-1.87, -0.09)	Helmet NIV	-0.44 (-1.37, 0.5)	-0.27 (-1.24, 0.72)
HFNC	-0.54 (-0.82, -0.26)	0.44 (-0.5, 1.37)	HFNC	0.17 (-0.11, 0.45)
COT	-0.71 (-1.11, -0.31)	0.27 (-0.72, 1.24)	-0.17 (-0.45, 0.11)	COT
**Length of stay in hospital, days**				
	Face mask NIV	Helmet NIV	HFNC	COT
Face mask NIV	Face mask NIV	-2.44 (-8.35, 3.47)	-0.73 (-1.35, -0.1)	-1.09 (-2, -0.18)
Helmet NIV	2.44 (-3.47, 8.35)	Helmet NIV	1.71 (-4.24, 7.67)	1.35 (-4.63, 7.34)
HFNC	0.73 (0.1, 1.35)	-1.71 (-7.67, 4.24)	HFNC	-0.36 (-1.02, 0.3)
COT	1.09 (0.18, 2)	-1.35 (-7.34, 4.63)	0.36 (-0.3, 1.02)	COT
**Respiratory rate, min**				
	Face mask NIV	Helmet NIV	HFNC	COT
Face mask NIV	Face mask NIV	-0.82 (-1.87, 0.23)	0.27 (-0.52, 1.06)	0.21 (-0.65, 1.06)
Helmet NIV	0.82 (-0.23, 1.87)	Helmet NIV	1.09 (-0.23, 2.41)	1.02 (-0.33, 2.38)
HFNC	-0.27 (-1.06, 0.52)	-1.09 (-2.41, 0.23)	HFNC	-0.07 (-0.39, 0.26)
COT	-0.21 (-1.06, 0.65)	-1.02 (-2.38, 0.33)	0.07 (-0.26, 0.39)	COT
**PaCO**_**2**_ **, mmHg**				
	Face mask NIV	Helmet NIV	HFNC	COT
Face mask NIV	Face mask NIV	6.51 (4.04, 8.96)	0.1 (-1.37, 1.56)	0.73 (-1.3, 2.76)
Helmet NIV	-6.51 (-8.96, -4.04)	Helmet NIV	-6.41 (-9.26, -3.55)	-5.77 (-8.96, -2.59)
HFNC	-0.1 (-1.56, 1.37)	6.41 (3.55, 9.26)	HFNC	0.64 (-0.76, 2.03)
COT	-0.73 (-2.76, 1.3)	5.77 (2.59, 8.96)	-0.64 (-2.03, 0.76)	COT

COT: Conventional oxygen therapy; HFNC: High flow nasal cannula; NIV: Non-invasive ventilation; PaCO_2_: Arterial carbon dioxide partial pressure.

**Table 3:** Rank probabilities of different non-invasive respiratory support methods for each outcome.

**Outcomes**	**Rank probabilities**	**Rank probabilities**	**Rank probabilities**	**Rank probabilities**
**Treatment failure**				
	[1]	[2]	[3]	
Face mask NIV	0.001215	0.011895	0.98689	
HFNC	0.075875	0.91541	0.008715	
COT	0.92291	0.072695	0.004395	
**Intubation**				
	[1]	[2]	[3]	[4]
Face mask NIV	0.4967	0.429945	0.07119	0.002165
Helmet NIV	0.028135	0.032075	0.62676	0.31303
HFNC	0.3847	0.513805	0.09642	0.005075
COT	0.090465	0.024175	0.20563	0.67973
**All-cause mortality**				
	[1]	[2]	[3]	[4]
Face mask NIV	0.17408	0.37199	0.32952	0.12441
Helmet NIV	0.39203	0.093305	0.115885	0.39878
HFNC	0.159985	0.37037	0.348145	0.1215
COT	0.273905	0.164335	0.20645	0.35531
**Complications**				
	[1]	[2]	[3]	
Face mask NIV	0.96499	0.03499	0.00002	
Helmet NIV	0.03471	0.42973	0.53556	
HFNC	0.0003	0.53528	0.46442	
**Dyspnea score**				
	[1]	[2]	[3]	[4]
Face mask NIV	0	0.00004	0.0161	0.98386
Helmet NIV	0.701455	0.123565	0.15916	0.01582
HFNC	0.02128	0.25602	0.72262	0.00008
COT	0.277265	0.620375	0.10212	0.00024
**Length of stay in hospital, days**				
	[1]	[2]	[3]	[4]
Face mask NIV	0.775090	0.218930	0.005435	0.000545
Helmet NIV	0.209815	0.075580	0.047365	0.667240
HFNC	0.008505	0.608195	0.343735	0.039565
COT	0.006590	0.097295	0.603465	0.292650
**Respiratory rate, min**				
	[1]	[2]	[3]	[4]
Face mask NIV	0.212090	0.124905	0.621265	0.041740
Helmet NIV	0.027415	0.028060	0.048315	0.896210
HFNC	0.481615	0.400870	0.104055	0.013460
COT	0.278880	0.446165	0.226365	0.048590
**PaCO**_**2**_ **, mmHg**				
	[1]	[2]	[3]	[4]
Face mask NIV	0.00000	0.20423	0.27957	0.51620
Helmet NIV	0.99984	0.00016	0.00000	0.00000
HFNC	0.00000	0.10186	0.53260	0.36554
COT	0.00016	0.69375	0.18783	0.11826

COT: Conventional oxygen therapy; HFNC: High flow nasal cannula; NIV: Non-invasive ventilation; PaCO_2_: Arterial carbon dioxide partial pressure.

### Intubation

**Figure 3:**
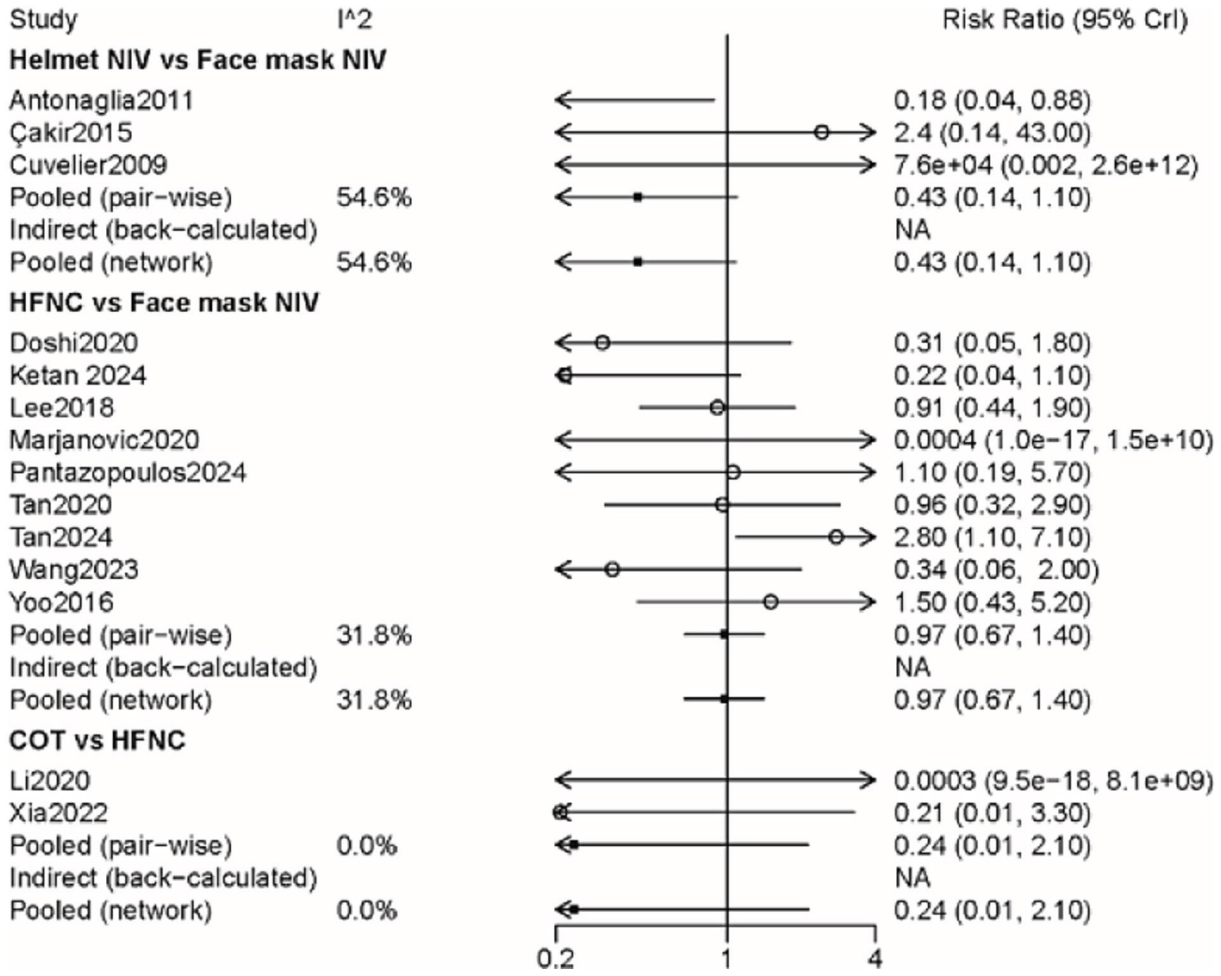
Forest plot on meta-analysis of intubation rates after treatment of AHRF with different noninvasive respiratory support methods.

A total of 14 studies involving 1,541 patients were included, with interventions involving four different methods (Supplementary Figure 3). There were no significant differences among these approaches, as shown by the forest plot (Figure 3 and Table 2). According to the rank probabilities, the COT group had the smallest probability of intubation among the four methods (Table 3).

### All-cause mortality

**Figure 4:**
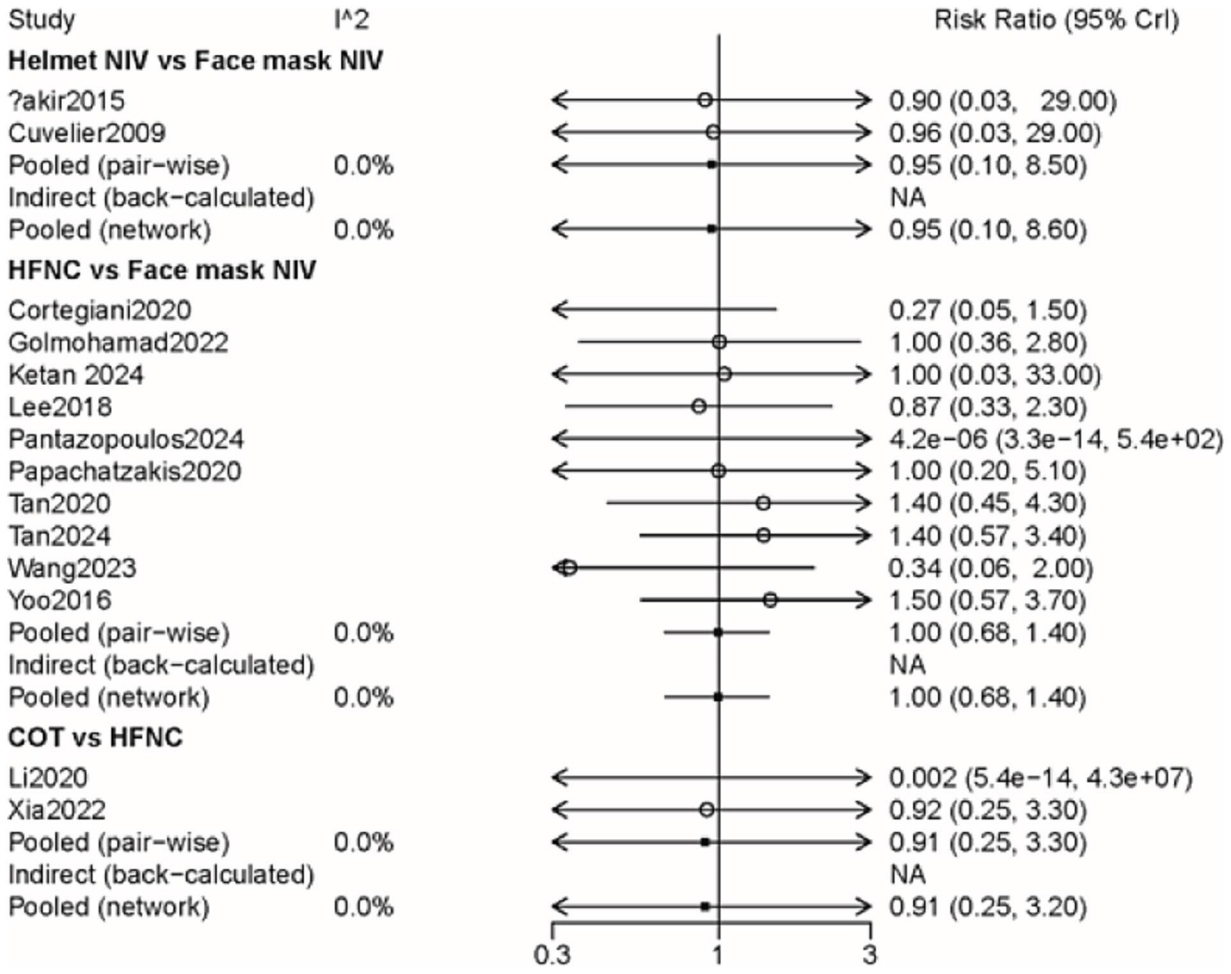
Forest plot on meta-analysis of all-cause mortality after treatment of AHRF with different noninvasive respiratory support methods.

A total of 14 studies involving 1,592 patients were included, with interventions encompassing four distinct methods (Supplementary Figure 4). As shown in the forest plot (Figure 4), there were no significant differences between the methods (Table 2). From the rank probabilities, it can be observed that among the four methods, the COT group had the lowest probability of all-cause mortality (Table 3).

### Dyspnea score

Six studies with 865 patients assessed dyspnea scores, and four interventions were compared (Supplementary Figure 5). Table 4 showed that the MD and 95% CI for helmet NIV versus face mask NIV was 0.98 (0.083, 1.90), and for HFNC versus face mask NIV was 0.54 (0.26, 0.82). The league table results presented in Table 2 indicated that the dyspnea scores for the helmet NIV group (WMD: 1.42, 95% CI: 1.06, 1.93), COT group (WMD: 0.54, 95% CI: 0.26, 0.82), and HFNC group (WMD: 0.71, 95% CI: 0.31, 1.11) were all higher than those for the face mask NIV group. Additionally, the rank probabilities also demonstrated that the face mask NIV group had the lowest dyspnea scores.

**Table 4:** The pooled results of the meta-analysis concerning dyspnea score, LOS, respiratory rate, PaCO_2_.

Variables	Mean difference (95% CI)	*I*^2^
Dyspnea score		
Helmet NIV vs. face mask NIV		
Pisani2015	0.98 (0.08, 1.90)	
Pooled (pair-wise)	NA	
Indirect (back-calculated)	0.98 (0.08, 1.90)	
Pooled (network)	0.98 (0.08, 1.90)	
HFNC vs. face mask NIV		
Cortegiani2020	0.0004 (−0.88, 0.88)	
Doshi2020	−0.37 (−1.50, 0.72)	
Tan2020	0.65 (0.15, 1.10)	
Tan2024	0.70 (0.31, 1.10)	
Pooled (pair-wise)	0.54 (0.26, 0.82)	39.3%
Indirect (back-calculated)	NA	
Pooled (network)	0.54 (0.26, 0.82)	39.3%
COT vs. HFNC		
Xia2022	0.17 (−0.11, 0.45)	
Pooled (pair-wise)	0.17 (−0.11, 0.45)	
Indirect (back-calculated)	NA	
Pooled (network)	0.17 (−0.11, 0.45)	
LOS		
Helmet NIV vs. face mask NIV		
Cuvelier2009	−2.50 (−8.40, 3.50)	
Pooled (pair-wise)	−2.40 (−8.40, 3.50)	
Indirect (back-calculated)	NA	
Pooled (network)	−2.40 (−8.30, 3.40)	
HFNC vs. face mask NIV		
Cong2019	−0.26 (−2.20, 1.70)	
Cortegiani2020	0.21 (−2.70, 3.10)	
Ketan2024	−0.93 (−2.50, 0.66)	
Papachatzakis2020	0.54 (−5.20, 6.20)	
Tan2020	−1.30 (−2.80, 0.14)	
Tan2024	−0.65 (−1.50, 0.24)	
Pooled (pair-wise)	−0.72 (−1.4, −0.09)	0%
Indirect (back-calculated)	NA	
Pooled (network)	−0.72 (−1.40, −0.10)	0%
COT vs. HFNC		
Li2020	0.70 (−0.38, 1.80)	
Xia2022	−1.00 (−1.8, −0.16)	
Pooled (pair-wise)	−0.36 (−1.00, 0.29)	83.3%
Indirect (back-calculated)	NA	
Pooled (network)	−0.36 (−1.00, 0.29)	83.3%
Respiratory rate		
Helmet NIV vs. face mask NIV		
Antonaglia2011	−0.82 (−2.00, 0.35)	
Pisani2015	−0.83 (−3.20, 1.60)	
Pooled (pair-wise)	−0.82 (−1.90, 0.23)	0%
Indirect (back-calculated)	NA	
Pooled (network)	−0.82 (−1.90, 0.23)	0%
HFNC vs. face mask NIV		
Cortegiani2020	−1.0 (−2.80, 0.77)	
Doshi2020	−0.27 (−2.60, 2.00)	
Marjanovic2020	5.00 (1.20, 8.80)	
Papachatzakis2020	−1.60 (−4.10, 0.94)	
Tan2020	−1.70 (−4.40, 1.00)	
Tan2024	1.30 (0.13, 2.50)	
Pooled (pair-wise)	0.28 (−0.52, 1.10)	67.0%
Indirect (back-calculated)	NA	
Pooled (network)	0.28 (−0.52, 1.10)	67.0%
COT vs. HFNC		
Li2020	0.40 (−0.12, 0.93)	
Xia2022	−0.35 (−0.76, 0.06)	
Pooled (pair-wise)	−0.07 (−0.39, 0.26)	79.6%
Indirect (back-calculated)	NA	
Pooled (network)	−0.07 (−0.39, 0.26)	79.6%
PaCO_2_		
Helmet NIV vs. face mask NIV		
Antonaglia2011	6.50 (4.10, 9.00)	
Pooled (pair-wise)	6.50 (4.10, 9.00)	
Indirect (back-calculated)	NA	
Pooled (network)	6.50 (4.00, 9.00)	
HFNC vs. face mask NIV		
Cortegiani2020	5.90 (−0.11, 12.00)	
Doshi2020	−8.00 (−16.00, −0.42)	
Marjanovic2020	−6.90 (−15.00, 0.88)	
Papachatzakis2020	−8.70 (−16.00, −1.40)	
Tan2020	0.35 (−2.20, 2.90)	
Tan2024	1.70 (−0.67, 4.10)	
Pooled (pair-wise)	0.10 (−1.40, 1.60)	68.0%
Indirect (back-calculated)	NA	
Pooled (network)	0.09 (−1.40, 1.60)	68.0%
COT vs. HFNC		
Li2020	2.30 (0.07, 4.50)	
Xia2022	−0.41 (−2.20, 1.40)	71.0%
Pooled (pair-wise)	0.64 (−0.75, 2.00)	
Indirect (back-calculated)	NA	
Pooled (network)	0.63 (−0.75, 2.00)	71.0%

COT, conventional oxygen therapy; HFNC, high flow nasal cannula; NIV, non-invasive ventilation; PaCO_2_, arterial carbon dioxide partial pressure.

### Length of stay in hospital

In total, 1,344 efficacy assessments from nine publications were included to compare the length of stay in the hospital among the four methods (Supplementary Figure 6). The MD and 95% CI for HFNC versus face mask NIV was −0.72 (−1.4, −0.098), with all other outcomes being statistically insignificant (Table 4). Table 2 indicated that the length of hospital stays for face mask NIV was longer than that for HFNC (WMD: −0.73, 95% CI: −1.35, −0.10) and COT (WMD: −1.09, 95% CI: −2.00, −0.18). The rank probabilities concluded that COT possessed the shortest hospital stay.

### Respiratory rate

A total of 14 studies involving 1,592 patients were included, with interventions comprising four distinct methods (Supplementary Figure 7). No significant differences among the methods was observed (Table 4). The rank probability table revealed that among the four methods, the helmet NIV group had the best treatment effect on respiratory rate (Table 3).

### PaCO_2_

Ten articles with 1,380 patients evaluated PaCO_2_, and four interventions were compared (Supplementary Figure 8). Aside from the comparison between helmet NIV and face mask NIV groups which showed statistical significance (WMD: 6.50, 95% CI: 4.00, 9.00), other group comparisons did not have statistical significance (Table 4). The PaCO_2_ levels in the helmet NIV group were higher than those in the face mask NIV group (WMD: 6.51, 95% CI: 4.04, 8.96). The PaCO_2_ of HFNC (WMD: −6.41, 95% CI: −9.26, −3.55) and COT groups (WMD: −5.77, 95% CI: −8.96, −2.59) were lower than helmet NIV group (Table 2). The rank probability table demonstrated that the face mask NIV group possessed the lowest PaCO_2_ levels (Table 3).

### Complications

**Figure 5:**
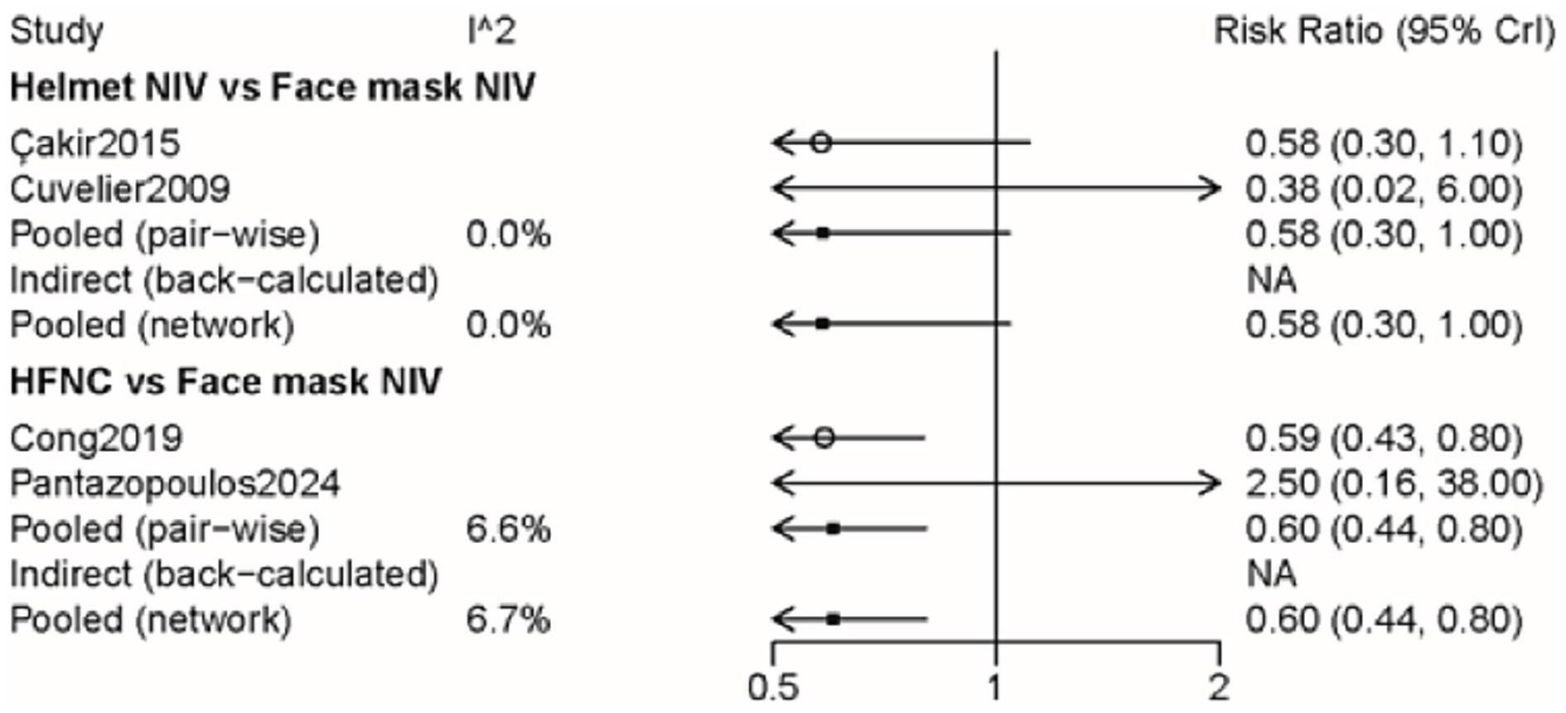
Forest plot on meta-analysis of complications after treatment of AHRF with different noninvasive respiratory support methods.

The impact of three approaches on complications was assessed through four pieces of literature with 355 participants (Supplementary Figure 9). The incidence of complications in the HFNC group was lower than that in the face mask NIV group (RR: 0.60, 95% CI: 0.44, 0.80) (Figure 5 and Table 2). The rank probability table indicated that the helmet NIV group had the lowest probability of complications (Table 3).

## Discussion

In this NMA of 19 articles including 2,022 participants, the impact of different non-invasive respiratory support modalities on treatment failure, intubation, all-cause mortality, dyspnea score, length of stay in hospital, complications, and the effectiveness in improving PaCO_2_ and respiratory rate in patients with AHRF was compared. The results indicated that AHRF patients treated with face mask NIV had a lower likelihood of treatment failure compared to those treated with HFNC and COT, and that face mask NIV was significantly more effective in improving dyspnea scores and PaCO_2_ levels than the other three methods. However, patients treated with face mask NIV had a longer hospital stay compared to those treated with COT and HFNC, and the probability of complications with face mask NIV might be higher than with HFNC. Regarding intubation and all-cause mortality outcomes, the rank probability table suggested that COT might offer superior treatment effects compared to the other three methods. In terms of improving respiratory rate in AHRF patients, helmet NIV may be more effective than the remaining methods.

In recent years, with the continuous advancement of clinical practice, the application of NIV has been widely promoted as an effective strategy for managing AHRF (11, 23). Face mask NIV, applied in accordance with the patient’s facial contours, is a classic method of NIV and has become a fundamental treatment for AHRF patients (24, 25). In our study, we compared the impact of face mask NIV with HFNC and COT on treatment failure rates and found that face mask NIV may be associated with a potentially lower probability of treatment failure in AHRF patients. Xu’s study published a conclusion similar to ours (3). However, in the study by Pantazopoulos et al. (26), it was not found that face mask NIV had a superior effect on treatment failure compared to HFNC; instead, it revealed a similar impact capacity between the two. Another study conducted in patients with coronavirus disease 19-related acute respiratory failure also considered the probabilities of treatment failure to be comparable among the aforementioned three methods (27). Additionally, we also observed that face mask NIV demonstrated superior effects on dyspnea scores and PaCO_2_ compared to helmet NIV, HFNC, and COT. Özlem et al. (28) also found that the decline in PaCO_2_ was slower in the helmet NIV group than in the face mask NIV group. NIV has been demonstrated to positively enhance inspiratory tidal volume and effectively increase pH levels while reducing PaCO_2_ (3, 29). Although both face mask NIV and helmet NIV belong to the NIV modalities, they yield different outcomes in the improvement of PaCO_2_. This discrepancy may stem from the lower pressurization rate and trigger performance efficiency of helmets compared to masks in terms of effectiveness (30). Face mask NIV is not without its drawbacks. Numerous studies have found that at higher airway pressures, the face mask interface may have poor tolerance and can be associated with air leaks, which can impair oxygenation (31, 32). In addition, the arbitrary 20% increase in pressure under PSV and PEEP in the helmet group may not be sufficient to compensate for the energy dissipated against the helmet wall or to effectively establish a rapid pressure gradient. A previous study reported that when a helmet and a mask are used with identical pressure settings, the helmet induces a significantly greater inspiratory muscle effort. However, this increased effort is effectively eliminated by increasing both PSV and PEEP by 50% (33). Alternatively, for patients with claustrophobia or those prone to facial pressure ulcers, the ability to tolerate a face mask is weaker (34, 35). Overall, the application of face mask NIV in the treatment of patients with AHRF requires the development of a treatment plan that takes into account the individual’s specific circumstances. Given the controversial nature of the aforementioned content, further research is still needed in the future to verify these conclusions.

Regarding the impact on the length of stay in hospital for patients with AHRF, the present survey indicated that the duration of hospitalization for patients treated with face mask NIV was longer compared to those treated with HFNC and COT. This may be attributed to the fact that patients receiving COT and HFNC experience greater comfort and tolerance compared to those on face mask NIV, which could potentially facilitate more rapid recovery and expedite discharge from the hospital (36, 37). Likewise, the findings by Xu et al. (3), who compared NIV and HFNC, found that HFNC could significantly reduce the length of hospital stay compared to NIV. Also, HFNC possesses numerous physiological advantages, which have contributed to its widespread adoption in the treatment of adult respiratory failure in recent years (38). It efficiently delivers humidified and heated gas to the airway (39). Furthermore, the high flow rate and warm, humidified gas provided by HFNC can reduce inspiratory resistance while increasing expiratory resistance (40). High flow rates also effectively wash out CO_2_, decrease anatomical dead space, and enhance minute ventilation, thereby improving gas exchange (41). COT had similar effect on oxygenation improvement and CO_2_ clearance with HFNC in patients with acute compensated hypercapnic respiratory failure (3). Some studies have also explored the physiological effects of HFNC in patients of COPD. HFNC could decrease the neuroventilatory drive and work of breathing in patients with COPD compared with COT.25 O.

Nonetheless, in Tan’s et al. (42) study, which compared the hospital stay between HFNC and NIV, no significant statistical differences were observed. Furthermore, regarding the incidence of complications, the research observed that the probability of developing complications in AHRF patients treated with HFNC may be significantly lower than in those treated with face mask NIV. It is acknowledged that HFNC is a device that delivers up to 100% inspired oxygen through a nasal cannula (43, 44). Whereas face mask NIV is a device that makes direct contact with the skin and is relatively tight, face masks tend to possess a higher incidence of adverse events (45, 46). Therefore, the above-observed results for the incidence of complications are reasonable.

In the final observation of the impact of different respiratory support methods on intubation, all-cause mortality, and respiratory rate in patients with AHRF, no significant statistical differences were observed in comparisons. The occurrence of this phenomenon may be attributed to the limited sample size included in this study, which led to the failure to receive anticipated analyzing outcomes. However, the rank probability table revealed that patients with AHRF treated with COT may have the lowest probability of intubation and death. Considering previous studies conducted in populations with respiratory failure, it has been indicated that the choice of treatment type and respiratory support strategy should be based on the severity of the disease (47, 48). We reasonably speculated that the observed outcome may be largely due to the fact that AHRF patients treated with COT might had less severe conditions compared to others, and thus could achieve recovery after treatment without the need for intubation or facing mortality. However, due to limitations in the data, it was not possible to further distinguish AHRF patients based on the severity of their condition or the duration of the disease, which precluded the use of subgroup analysis to validate the above hypothesis. Therefore, future study designs could consider the severity of patients’ conditions as an important factor. What’s more, in terms of improving respiratory rate for AHRF patients, helmet NIV had the highest probability of being ranked first, suggesting that helmet NIV may have a promising effect on improving respiratory rate. Although Hong et al. (25) conducted their study in the population of acute respiratory failure, they arrived at similar conclusions to ours, indicating that helmet NIV had significant effects in improving respiratory rate. These results suggested that helmet NIV could be integrated into clinical practice for the treatment of respiratory rate in patients with AHRF in the future.

This study possesses certain strengths; through NMA, we compared the therapeutic effects of multiple non-invasive respiratory support methods on patients with AHRF, rather than just a single non-invasive respiratory support method against conventional treatment. This approach more comprehensively assessed the relative efficacy of different interventions. For selected patients with AHRF, face mask NIV might be considered for potential strategy that had a lower likelihood of treatment failure compared to those treated with HFNC and COT, and that face mask NIV was significantly more effective in improving dyspnea scores and PaCO_2_ levels than the other three methods. On the other hand, due to the longer hospital stay, and the probability of complications with face mask NIV, respective preventions should be performed for patients receiving this treatment. However, similar to all research endeavors, our study had its inherent limitations. Firstly, the quality of some outcomes was relatively low. This may introduce a certain level of bias risk, diminish the confidence in the effect estimates, and suggest the need for further research to substantiate the findings of this survey. Secondly, there was a limited number of studies for certain interventions, affecting the stability of the results and thus reducing their credibility. Thirdly, there was heterogeneity observed in some of the analyses. This may be caused by the differences in research subjects, intervention measures and outcome indicators in different studies. High heterogeneity may decrease the interpretability and generalization of the results. Thus, the replication studies, heterogeneous study design, and longitudinal studies are needed to further validate the findings in this study. Lastly, since our study also included a small number of cohort studies, it was inevitably subject to inherent biases within the studies, and the presence of confounding factors may also influence the outcomes to varying degrees. In summary, future prospective, large-scale, and higher-level evidence studies are needed to address the aforementioned limitations, to find superior non-invasive respiratory support methods for treating AHRF, and to strive for the overall health of patients with AHRF.

## Conclusion

Our study found that face mask NIV may be superior to other methods in terms of treatment failure, dyspnea score, and improvement of PaCO_2_ in AHRF patients. However, face mask NIV did not show better improvement effects on other outcomes. Future large-scale RCT studies with higher levels of evidence are still needed to determine the optimal non-invasive respiratory support method for treating AHRF patients.

## Data Availability

The raw data supporting the conclusions of this article will be made available by the authors, without undue reservation.

## Supplementary material

SUPPLEMENTARY FIGURE 1 The results of risk of bias. **(a)** Risk of bias graph. **(b)** Risk of bias summary.

SUPPLEMENTARY FIGURE 2 A network evidence plot on meta-analysis of treatment failure after treatment of AHRF with different noninvasive respiratory support methods.

SUPPLEMENTARY FIGURE 3 A network evidence plot on meta-analysis of intubation rates after treatment of AHRF with different noninvasive respiratory support methods.

SUPPLEMENTARY FIGURE 4 A network evidence plot on meta-analysis of all-cause mortality after treatment of AHRF with different noninvasive respiratory support methods.

SUPPLEMENTARY FIGURE 5 A network evidence plot on meta-analysis of dyspnea score after treatment of AHRF with different noninvasive respiratory support methods.

SUPPLEMENTARY FIGURE 6 A network evidence plot on meta-analysis for length of stay in hospital after treatment of AHRF with different noninvasive respiratory support methods.

SUPPLEMENTARY FIGURE 7 A network evidence plot on meta-analysis of respiratory rate after treatment of AHRF with different noninvasive respiratory support methods.

SUPPLEMENTARY FIGURE 8 A network evidence plot on meta-analysis of PaCO_2_ after treatment of AHRF with different noninvasive respiratory support methods.

SUPPLEMENTARY FIGURE 9 A network evidence plot on meta-analysis of complications after treatment of AHRF with different noninvasive respiratory support methods.
