# Supplementary figures and images for: A comparative analysis of non-invasive respiratory support modalities in the treatment of acute hypercapnic respiratory failure: a network meta-analysis
Source: Front Med (Lausanne). 2025 Jul 8;12:1594128. doi: 10.3389/fmed.2025.1594128 (PMC12279498; doi:10.3389/fmed.2025.1594128)

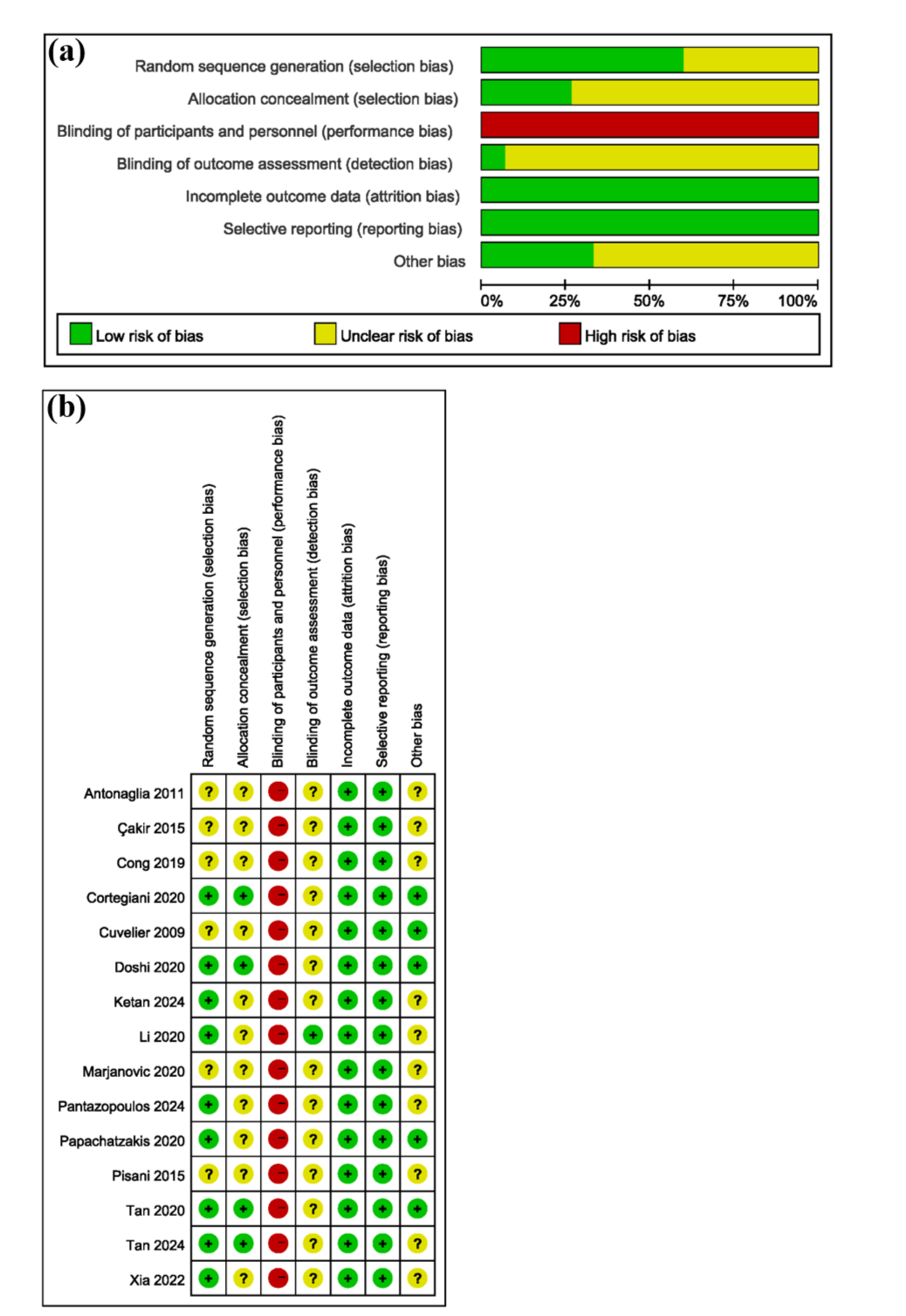

Supplement: SUPPLEMENTARY FIGURE 1 — The results of risk of bias. (a) Risk of bias graph. (b) Risk of bias summary. [file Image_1.tif]

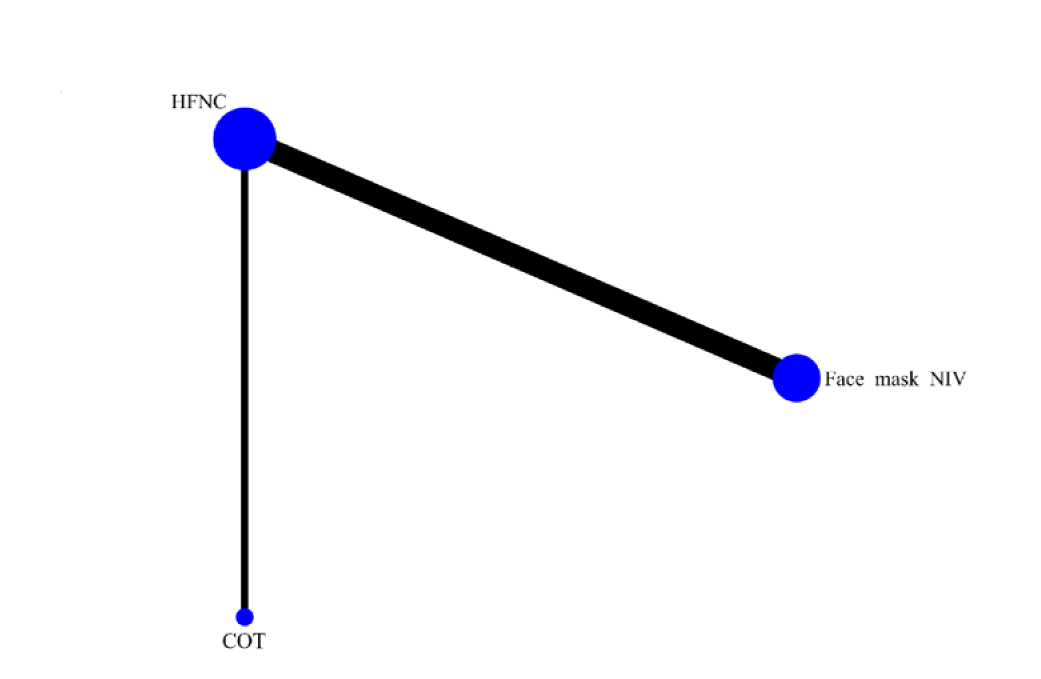

Supplement: SUPPLEMENTARY FIGURE 2 — A network evidence plot on meta-analysis of treatment failure after treatment of AHRF with different noninvasive respiratory support methods. [file Image_2.tif]

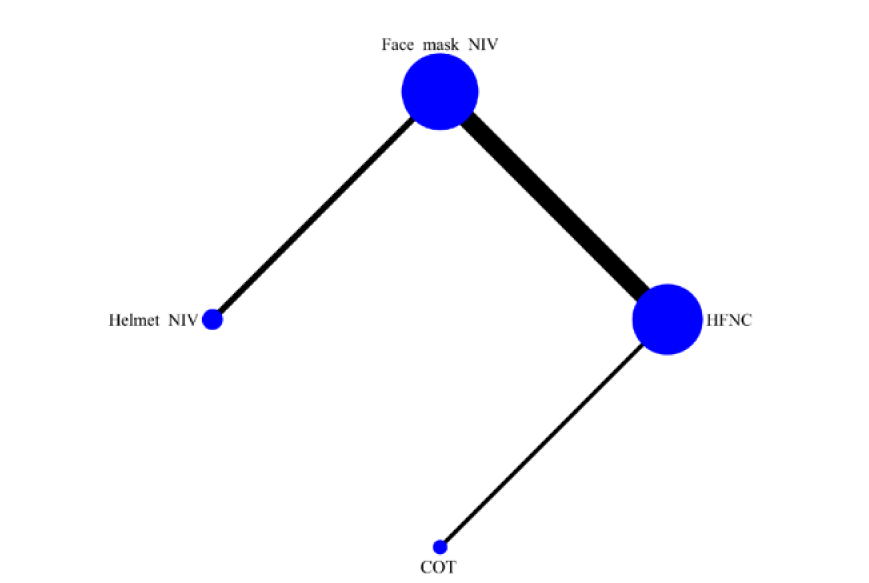

Supplement: SUPPLEMENTARY FIGURE 3 — A network evidence plot on meta-analysis of intubation rates after treatment of AHRF with different noninvasive respiratory support methods. [file Image_3.tif]

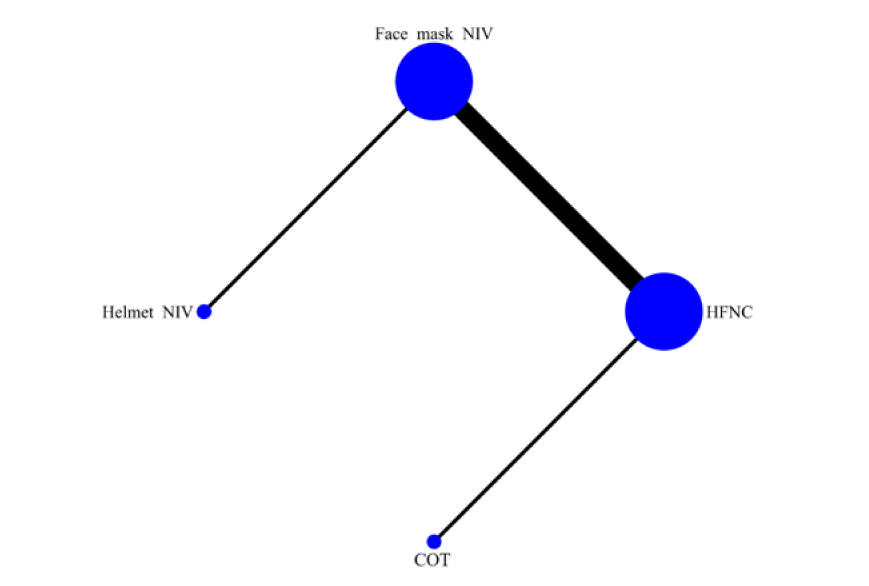

Supplement: SUPPLEMENTARY FIGURE 4 — A network evidence plot on meta-analysis of all-cause mortality after treatment of AHRF with different noninvasive respiratory support methods. [file Image_4.tif]

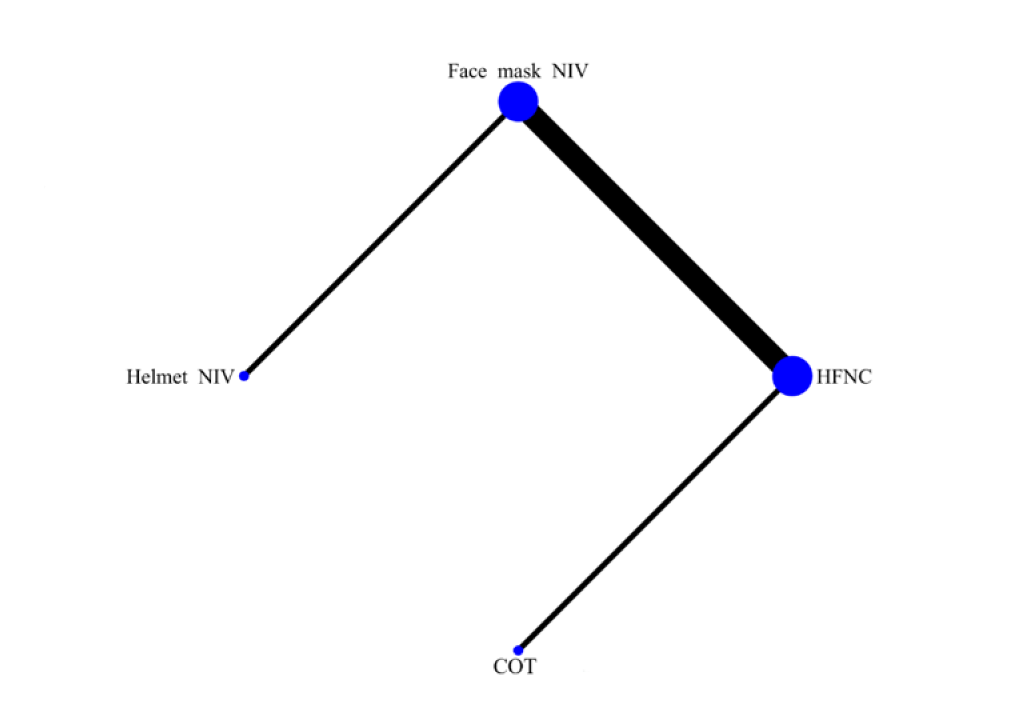

Supplement: SUPPLEMENTARY FIGURE 5 — A network evidence plot on meta-analysis of dyspnea score after treatment of AHRF with different noninvasive respiratory support methods. [file Image_5.tif]

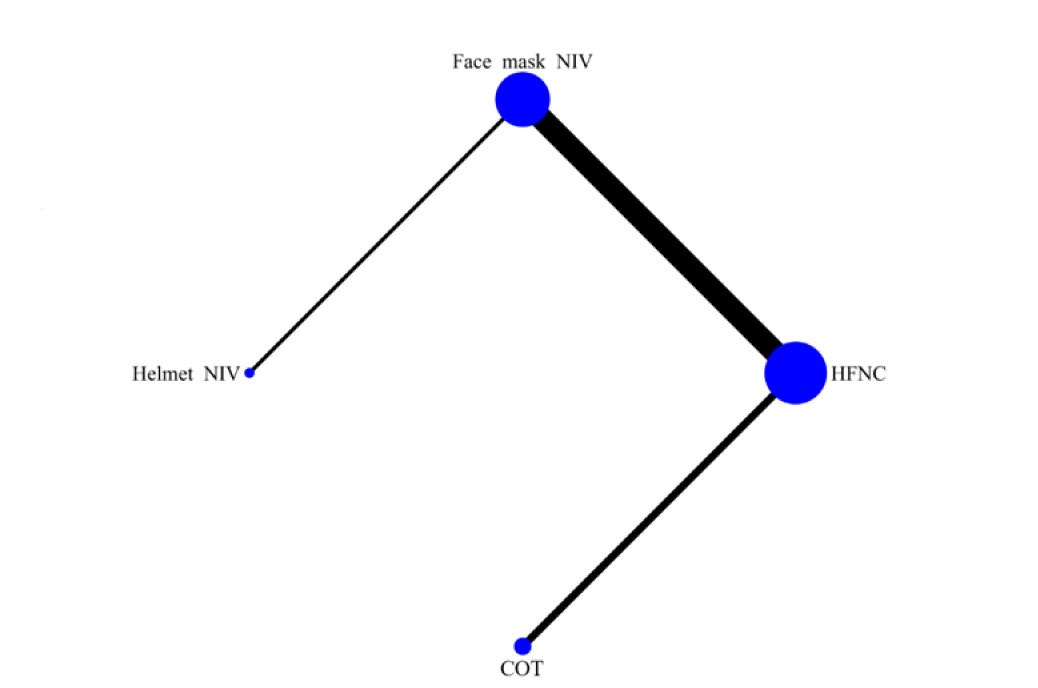

Supplement: SUPPLEMENTARY FIGURE 6 — A network evidence plot on meta-analysis for length of stay in hospital after treatment of AHRF with different noninvasive respiratory support methods. [file Image_6.tif]

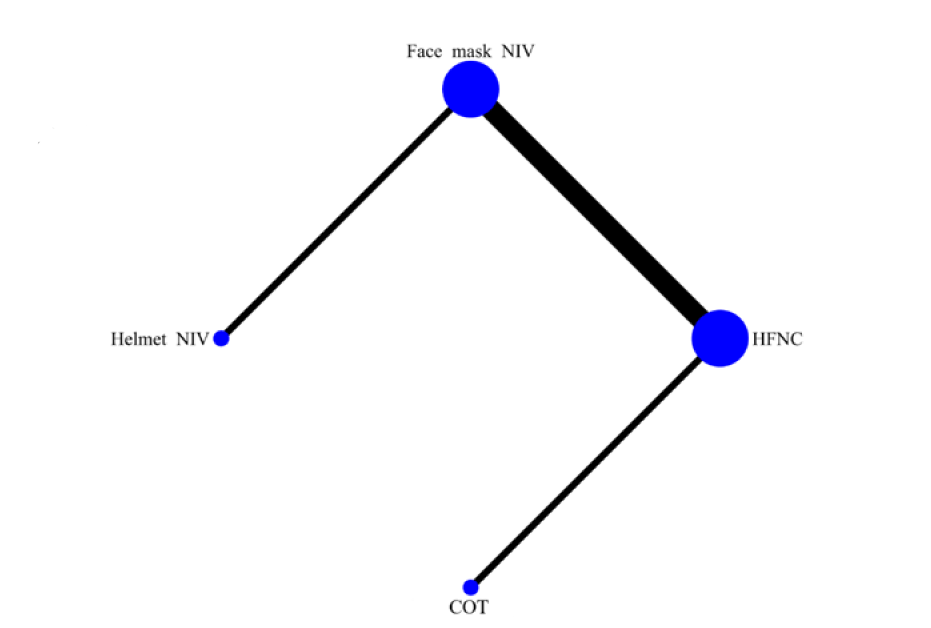

Supplement: SUPPLEMENTARY FIGURE 7 — A network evidence plot on meta-analysis of respiratory rate after treatment of AHRF with different noninvasive respiratory support methods. [file Image_7.tif]

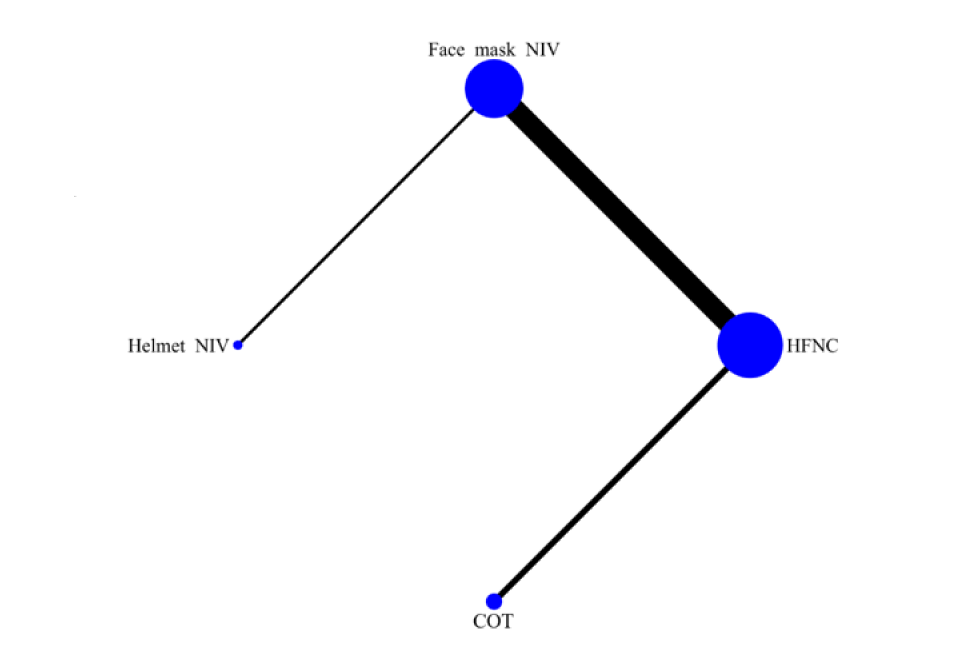

Supplement: SUPPLEMENTARY FIGURE 8 — A network evidence plot on meta-analysis of PaCO2 after treatment of AHRF with different noninvasive respiratory support methods. [file Image_8.tif]

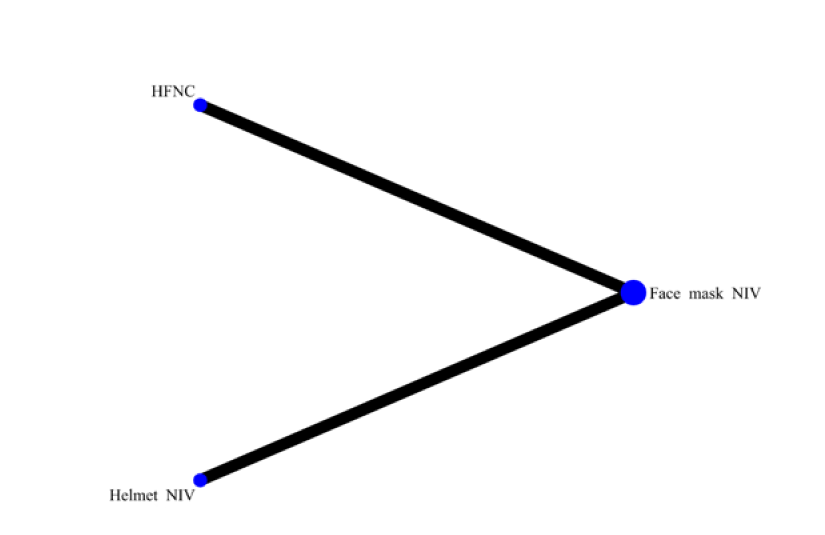

Supplement: SUPPLEMENTARY FIGURE 9 — A network evidence plot on meta-analysis of complications after treatment of AHRF with different noninvasive respiratory support methods. [file Image_9.TIF]
