# Supplementary material for: A comparative analysis of non-invasive respiratory support modalities in the treatment of acute hypercapnic respiratory failure: a network meta-analysis
Source: Front Med (Lausanne). 2025 Jul 8;12:1594128. doi: 10.3389/fmed.2025.1594128 (PMC12279498; doi:10.3389/fmed.2025.1594128)
Supplement: Supplementary file 10 [file Supplementary_file_1.docx]

**Included literature**

1. Cuvelier A, Pujol W, Pramil S, Molano LC, Viacroze C, Muir JF: **Cephalic versus oronasal mask for noninvasive ventilation in acute hypercapnic respiratory failure**. *Intensive care medicine* 2009, **35**(3):519-526.

2. Yoo JW, Synn A, Huh JW, Hong SB, Koh Y, Lim CM: **Clinical efficacy of high-flow nasal cannula compared to noninvasive ventilation in patients with post-extubation respiratory failure**. *The Korean journal of internal medicine* 2016, **31**(1):82-88.

3. Özlem Ç G, Ali A, Fatma U, Mehtap T, Şaziye Ş: **Comparison of helmet and facial mask during noninvasive ventilation in patients with acute exacerbation of chronic obstructive pulmonary disease: a randomized controlled study**. *Turkish journal of medical sciences* 2015, **45**(3):600-606.

4. Antonaglia V, Ferluga M, Molino R, Lucangelo U, Peratoner A, Roman-Pognuz E, De Simoni L, Zin WA: **Comparison of noninvasive ventilation by sequential use of mask and helmet versus mask in acute exacerbation of chronic obstructive pulmonary disease: a preliminary study**. *Respiration; international review of thoracic diseases* 2011, **82**(2):148-154.

5. Tan D, Wang B, Cao P, Wang Y, Sun J, Geng P, Walline JH, Wang Y, Wang C: **High flow nasal cannula oxygen therapy versus non-invasive ventilation for acute exacerbations of chronic obstructive pulmonary disease with acute-moderate hypercapnic respiratory failure: a randomized controlled non-inferiority trial**. *Critical care (London, England)* 2024, **28**(1):250.

6. Lee MK, Choi J, Park B, Kim B, Lee SJ, Kim SH, Yong SJ, Choi EH, Lee WY: **High flow nasal cannulae oxygen therapy in acute-moderate hypercapnic respiratory failure**. *The clinical respiratory journal* 2018, **12**(6):2046-2056.

7. Cortegiani A, Longhini F, Madotto F, Groff P, Scala R, Crimi C, Carlucci A, Bruni A, Garofalo E, Raineri SM *et al*: **High flow nasal therapy versus noninvasive ventilation as initial ventilatory strategy in COPD exacerbation: a multicenter non-inferiority randomized trial**. *Critical care (London, England)* 2020, **24**(1):692.

8. Li XY, Tang X, Wang R, Yuan X, Zhao Y, Wang L, Li HC, Chu HW, Li J, Mao WP *et al*: **High-Flow Nasal Cannula for Chronic Obstructive Pulmonary Disease with Acute Compensated Hypercapnic Respiratory Failure: A Randomized, Controlled Trial**. *International journal of chronic obstructive pulmonary disease* 2020, **15**:3051-3061.

9. Marjanovic N, Flacher A, Drouet L, Gouhinec AL, Said H, Vigneau JF, Chollet B, Lefebvre S, Sebbane M: **High-Flow Nasal Cannula in Early Emergency Department Management of Acute Hypercapnic Respiratory Failure Due to Cardiogenic Pulmonary Edema**. *Respiratory care* 2020, **65**(9):1241-1249.

10. Tan D, Walline JH, Ling B, Xu Y, Sun J, Wang B, Shan X, Wang Y, Cao P, Zhu Q *et al*: **High-flow nasal cannula oxygen therapy versus non-invasive ventilation for chronic obstructive pulmonary disease patients after extubation: a multicenter, randomized controlled trial**. *Critical care (London, England)* 2020, **24**(1):489.

11. Xia J, Gu S, Lei W, Zhang J, Wei H, Liu C, Zhang H, Lu R, Zhang L, Jiang M *et al*: **High-flow nasal cannula versus conventional oxygen therapy in acute COPD exacerbation with mild hypercapnia: a multicenter randomized controlled trial**. *Critical care (London, England)* 2022, **26**(1):109.

12. Wang M, Zhao F, Sun L, Liang Y, Yan W, Sun X, Zhou Q, He B: **High-Flow Nasal Cannula versus Noninvasive Ventilation in AECOPD Patients with Respiratory Acidosis: A Retrospective Propensity Score-Matched Study**. *Canadian respiratory journal* 2023, **2023**:6377441.

13. Papachatzakis Y, Nikolaidis PT, Kontogiannis S, Trakada G: **High-Flow Oxygen through Nasal Cannula vs. Non-Invasive Ventilation in Hypercapnic Respiratory Failure: A Randomized Clinical Trial**. *International journal of environmental research and public health* 2020, **17**(16).

14. Pantazopoulos I, Boutlas S, Mavrovounis G, Papalampidou A, Papagiannakis N, Kontou M, Bibaki E, Athanasiou N, Meletis G, Gourgoulianis K *et al*: **Nasal high flow or noninvasive ventilation? navigating hypercapnic COPD exacerbation treatment: A randomized noninferiority clinical trial**. *Respiratory medicine* 2024, **232**:107762.

15. Pisani L, Mega C, Vaschetto R, Bellone A, Scala R, Cosentini R, Musti M, Del Forno M, Grassi M, Fasano L *et al*: **Oronasal mask versus helmet in acute hypercapnic respiratory failure**. *The European respiratory journal* 2015, **45**(3):691-699.

16. Cong L, Zhou LN, Liu HN, Wang JR: **Outcomes of high-flow nasal cannula versus non-invasive positive pressure ventilation for patients with acute exacerbations of chronic obstructive pulmonary disease**. *INTERNATIONAL JOURNAL OF CLINICAL AND EXPERIMENTAL MEDICINE* 2019, **12**(8):10863-10867.

17. Ketan PS, Kumar R, Aj M, Ish P, Chakrabarti S, Gupta NK, Gupta N: **Post-extubation high-flow nasal cannula oxygen therapy versus non-invasive ventilation in chronic obstructive pulmonary disease with hypercapnic respiratory failure**. *Monaldi archives for chest disease = Archivio Monaldi per le malattie del torace* 2023, **94**(2).

18. Golmohamad A, Johnston R, Hay K, Tay G: **Safety and efficacy of high-flow nasal cannula therapy in acute hypercapnic respiratory failure: a retrospective audit**. *Internal medicine journal* 2022, **52**(2):259-264.

19. Doshi PB, Whittle JS, Dungan G, 2nd, Volakis LI, Bublewicz M, Kearney J, Miller TL, Dodge D, Harsch MR, DeBellis R *et al*: **The ventilatory effect of high velocity nasal insufflation compared to non-invasive positive-pressure ventilation in the treatment of hypercapneic respiratory failure: A subgroup analysis**. *Heart & lung : the journal of critical care* 2020, **49**(5):610-615.
