# Supplementary material for: A comparative analysis of non-invasive respiratory support modalities in the treatment of acute hypercapnic respiratory failure: a network meta-analysis
Source: Front Med (Lausanne). 2025 Jul 8;12:1594128. doi: 10.3389/fmed.2025.1594128 (PMC12279498; doi:10.3389/fmed.2025.1594128)
Supplement: Supplementary file 11 [file Table_1.docx]

**Table S1 The search strategy of meta-analysis**

| **Items** | **Contents** |
| --- | --- |
| Search terms | Noninvasive Ventilation, Noninvasive Ventilations, Ventilation, Noninvasive, Ventilations, Noninvasive, Non-Invasive Ventilation, Non-Invasive Ventilations, Ventilation, Non-Invasive, Ventilations, Non-Invasive, Non Invasive Ventilation, Non Invasive Ventilations, Ventilation, Non Invasive, Ventilations, Non Invasive, NIV, continuous positive airway pressure, CPAP, Airway Pressure Release Ventilation, APRV, continuous positive pressure ventilation, CPPV, Bilevel Positive Airway Pressure, bi level positive airway pressure, bi-level positive airway pressure, BiPAP, Biphasic Positive Airway Pressure, Helmet, Helmets  Masks, Mask  high flow, High-flow, Highflow, Oxygen, Nasal, humidification oxygen, Humidication oxygen, humidified oxygen, HFNC, HFOC, Optiflow, Opti-flow, Opti flow  Respiratory Insufficiency, Respiratory Failure, Failure, Respiratory, Respiratory Failures, Respiratory Depression, Ventilatory Depression, Depressions, Ventilatory, Hypercapnic Respiratory Failure, Failure, Hypercapnic Respiratory, Hypercapnic Respiratory Failures, Respiratory Failure, Hypercapnic, Acute Hypercapnic Respiratory Failure, Hypercapnic Acute Respiratory Failure, Hypercapnia, Hypercapnic, Hypercapneic, Acidotic, AT2RF |
| PubMed search form | (((((((((((((((((((((((((((((Noninvasive Ventilation[Title/Abstract]) OR (Noninvasive Ventilations[Title/Abstract])) OR (Ventilation, Noninvasive[Title/Abstract])) OR (Ventilations, Noninvasive[Title/Abstract])) OR (Non-Invasive Ventilation[Title/Abstract])) OR (Non-Invasive Ventilations[Title/Abstract])) OR (Ventilation, Non-Invasive[Title/Abstract])) OR (Ventilations, Non-Invasive[Title/Abstract])) OR (Non Invasive Ventilation[Title/Abstract])) OR (Non Invasive Ventilations[Title/Abstract])) OR (Ventilation, Non Invasive[Title/Abstract])) OR (Ventilations, Non Invasive[Title/Abstract])) OR (NIV[Title/Abstract])) OR (continuous positive airway pressure[Title/Abstract])) OR (CPAP[Title/Abstract])) OR (Airway Pressure Release Ventilation[Title/Abstract])) OR (APRV[Title/Abstract])) OR (continuous positive pressure ventilation[Title/Abstract])) OR (CPPV[Title/Abstract])) OR (Bilevel Positive Airway Pressure[Title/Abstract])) OR (bi level positive airway pressure[Title/Abstract])) OR (bi-level positive airway pressure[Title/Abstract])) OR (BiPAP[Title/Abstract])) OR (Biphasic Positive Airway Pressure[Title/Abstract])) OR (Helmet[Title/Abstract])) OR (Helmets[Title/Abstract])) OR (Masks[Title/Abstract])) OR (Mask[Title/Abstract])) OR (((((high flow[Title/Abstract]) OR (High-flow[Title/Abstract])) OR (Highflow[Title/Abstract])) AND ((Oxygen[Title/Abstract]) OR (Nasal[Title/Abstract]))) OR ((((((((humidification oxygen[Title/Abstract]) OR (Humidication oxygen[Title/Abstract])) OR (humidified oxygen[Title/Abstract])) OR (HFNC[Title/Abstract])) OR (HFOC[Title/Abstract])) OR (Optiflow[Title/Abstract])) OR (Opti-flow[Title/Abstract])) OR (Opti flow[Title/Abstract])))) AND ((((((((((((((((((Respiratory Insufficiency[Title/Abstract]) OR (Respiratory Failure[Title/Abstract])) OR (Failure, Respiratory[Title/Abstract])) OR (Respiratory Failures[Title/Abstract])) OR (Respiratory Depression[Title/Abstract])) OR (Ventilatory Depression[Title/Abstract])) OR (Depressions, Ventilatory[Title/Abstract])) OR (Hypercapnic Respiratory Failure[Title/Abstract])) OR (Failure, Hypercapnic Respiratory[Title/Abstract])) OR (Hypercapnic Respiratory Failures[Title/Abstract])) OR (Respiratory Failure, Hypercapnic[Title/Abstract])) OR (Acute Hypercapnic Respiratory Failure[Title/Abstract])) OR (Hypercapnic Acute Respiratory Failure[Title/Abstract])) OR (Hypercapnia[Title/Abstract])) OR (Hypercapnic[Title/Abstract])) OR (Hypercapneic[Title/Abstract])) OR (Acidotic[Title/Abstract])) OR (AT2RF[Title/Abstract])) |

Table S2 Literature quality assessment of cohort study

| **Study** | **Ⅰ** | **Ⅱ** | **Ⅲ** | **Ⅳ** | **Ⅴ** | **Ⅵ** | **Ⅶ** | **Ⅷ** | **Total** |
| --- | --- | --- | --- | --- | --- | --- | --- | --- | --- |
| Golmohamad 2022 | 0 | 1 | 1 | 1 | 2 | 1 | 0 | 0 | 6 |
| Lee 2018 | 0 | 1 | 1 | 1 | 2 | 1 | 0 | 1 | 7 |
| Wang 2023 | 0 | 1 | 1 | 1 | 2 | 1 | 0 | 0 | 6 |
| Yoo 2016 | 0 | 1 | 1 | 1 | 2 | 1 | 0 | 0 | 6 |

Notes: Numbers I-Ⅷ in heading signified: Ⅰ: Representatives of the exposed cohort; Ⅱ: Selection of the non-exposed cohort; Ⅲ: Ascertainment of exposure; Ⅳ: Demonstration that outcome of interest was present at the start of the study; Ⅴ: Comparability of cohorts on the basis of the design or analysis; Ⅵ: Assessment of the outcome; Ⅶ: Was follow-up long enough for outcomes to occur? Ⅷ: Adequacy of follow-up of cohorts.

**Table S3 GRADE score results for all evidence**

| **Certainty assessment** | | | | | | | **Certainty** | **Importance** |
| --- | --- | --- | --- | --- | --- | --- | --- | --- |
| **№ of studies** | **Study design** | **Risk of bias** | **Inconsistency** | **Indirectness** | **Imprecision** | **Other considerations** |  |  |
| **Treatment failure** | | | | | | | | |
| 8 | Non-randomised studies | Serious^a^ | Not serious | Not serious | Not serious | None | ⨁◯◯◯ Very low^a^ | IMPORTANT |
| **Intubation** | | | | | | | | |
| 14 | Non-randomised studies | Serious^a^ | Not serious | Not serious | Serious^b^ | None | ⨁◯◯◯ Very low^a,b^ | IMPORTANT |
| **All-cause mortality** | | | | | | | | |
| 14 | Non-randomised studies | Serious^a^ | Not serious | Not serious | Serious^b^ | None | ⨁◯◯◯ Very low^a,b^ | IMPORTANT |
| **Complications** | | | | | | | | |
| 4 | Randomised trials | Serious^a^ | Serious^c^ | Not serious | Not serious | None | ⨁⨁◯◯ Low^a,c^ | IMPORTANT |
| **Length of stay in hospital** | | | | | | | | |
| 9 | Randomised trials | Serious^a^ | Not serious | Not serious | Serious^b^ | None | ⨁⨁◯◯ Low^a,b^ | IMPORTANT |
| **Respiratory rate** | | | | | | | | |
| 10 | Randomised trials | Serious^a^ | Not serious | Not serious | Not serious | None | ⨁⨁⨁◯ Moderate^a^ | IMPORTANT |
| **PaCO_2_** | | | | | | | | |
| 10 | Randomised trials | Serious^a^ | Serious^c^ | Not serious | Not serious | None | ⨁⨁◯◯ Low^a,c^ | IMPORTANT |
| **Dyspnea score** | | | | | | | | |
| 6 | Randomised trials | Serious^a^ | Not serious | Not serious | Not serious | None | ⨁⨁⨁◯ Moderate^a^ | IMPORTANT |

Note: CI**:** Confidence interval; Explanations: a. No blinding was used; b. Wide confidence intervals; c. Point estimates were highly variable across studies.
